# Stabilization of intermediate spin states in mixed-valent diiron dichalcogenide complexes

**DOI:** 10.1038/s41557-021-00853-5

**Published:** 2022-01-20

**Authors:** Justin T. Henthorn, George E. Cutsail, Thomas Weyhermüller, Serena DeBeer

**Affiliations:** 1 Max Planck Institute for Chemical Energy Conversion, Mülheim an der Ruhr, Germany; 2 Institute for Inorganic Chemistry, University of Duisburg-Essen, Essen, Germany

**Keywords:** Bioinorganic chemistry, Inorganic chemistry, Physical chemistry

## Abstract

The electronic structure and ground spin states, *S*, observed for mixed-valent iron–sulfur dimers (Fe^II^-Fe^III^) are typically determined by the Heisenberg exchange interaction, *J*, that couples the magnetic interaction of the two metal centres either ferromagnetically (*J* > 0, *S* = 9/2) or antiferromagnetically (*J* < 0, *S* = 1/2). In the case of antiferromagnetically coupled iron centres, stabilization of the high-spin *S* = 9/2 ground state is also feasible through a Heisenberg double-exchange interaction, *B*, which lifts the degeneracy of the Heisenberg spin states. This theorem also predicts intermediate spin states for mixed-valent dimers, but those have so far remained elusive. Herein, we describe the structural, electron paramagnetic resonance and Mössbauer spectroscopic, and magnetic characterization of a series of mixed-valent complexes featuring [Fe_2_Q_2_]^+^ (Q = S^2–^, Se^2–^, Te^2–^), where the Se and Te complexes favour *S* = 3/2 spin states. The incorporation of heavier chalcogenides in this series reveals a delicate balance of antiferromagnetic coupling, Heisenberg double-exchange and vibronic coupling.

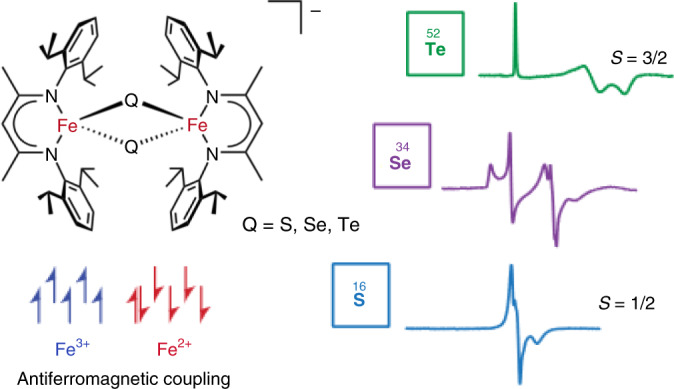

## Abstract (web summary)

Despite extensive investigations of mixed-valence complexes, molecules with intermediate spin states have remained elusive. Now, selenium- and tellurium-bridged mixed-valent iron dimers have been prepared in which a balance of Heisenberg exchange and double-exchange coupling of the unpaired electron, combined with moderate vibronic contributions, stabilizes *S* = 3/2 ground spin states.

## Main

Mixed-valent transition metal dimers play unique roles throughout chemistry, magnetism and biology. Iron–sulfur dimers in biology are integral to essential life processes and may be viewed as elementary building blocks to help understand the electronic structure of larger iron–sulfur cluster motifs^1,2^. The classic Heisenberg–Dirac–van Vleck Hamiltonian $-2J\hat S_1 \cdot \hat S_2$, where *J* is the Heisenberg exchange interaction and $\hat S$ is the spin operator, is used to describe the magnetic coupling of two magnetic centres and, for mixed-valent transition metal complexes, this Hamiltonian rationalizes the low- (*S* = 1/2) and high-spin (*S* = *n*/2) solutions. For reduced iron–sulfur dimers of Fe^II^-Fe^III^ valence, the isotropic Heisenberg exchange may be used to simply describe both the antiferromagnetically coupled *J* < 0, *S* = 1/2 and ferromagnetically coupled *J* > 0, *S* = 9/2 cases. The majority of synthetic and biological [Fe_2_S_2_]^+^ clusters possess *S* = 1/2 ground spin states arising from the antiferromagnetic coupling of the locally high-spin *d*^5^-*d*^6^ iron centres. However, there are limited examples, such as a mutant ferredoxin protein^3,4^ and [Fe_2_(OR)_2/3_]^3+/2+^ complexes^5–8^, that exhibit *S* = 9/2 ground spin states despite the fact that the iron centres remain antiferromagnetically coupled. The phenomenon of maximum spin in antiferromagnetically coupled mixed-valent metal centres is not limited to [Fe_2_(OR)_2/3_]^3+/2+^ clusters, but has also been observed in vanadium dimers^9,10^. The observed maximal spin states of these antiferromagnetically coupled dimers require further expansion of the Heisenberg–Dirac–van Vleck Hamiltonian.

The inclusion of the double-exchange interaction, *B*, lifts the degeneracy of the Heisenberg spin states into symmetric and antisymmetric pairs^11–13^, with the eigenvalues of the Heisenberg double-exchange Hamiltonian becoming:

1 $$E_ \pm = - JS(S + 1) \pm B(S + 1/2)$$

**Fig. 1:**
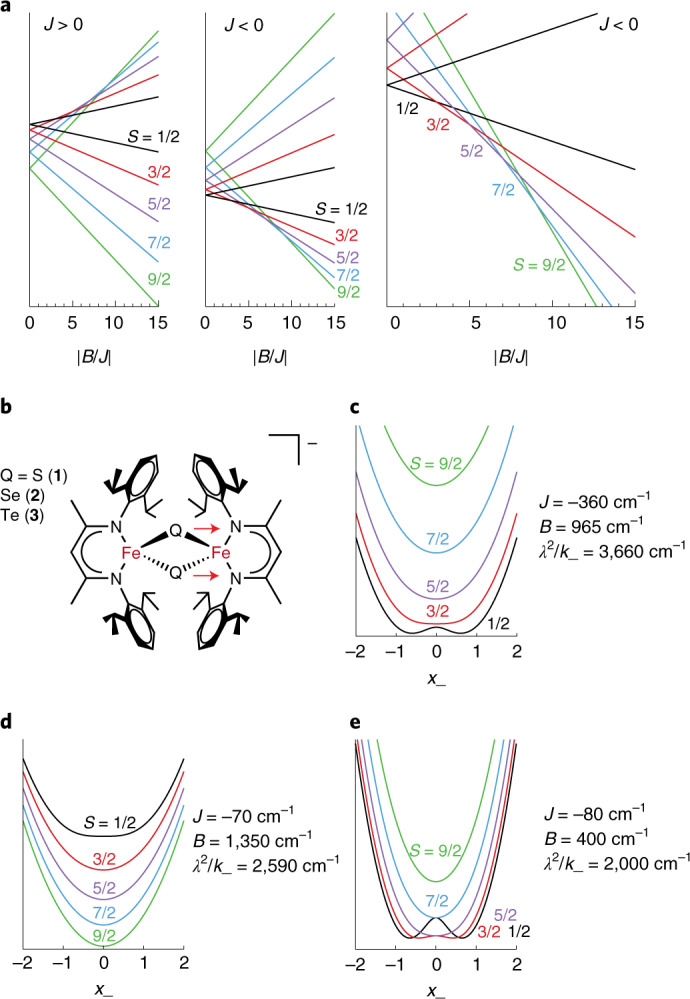
Heisenberg double-exchange spin-state energy diagrams of a *d*^5^-*d*^6^ mixed-valent dimer and the influence of vibronic coupling. **a**, Energy levels determined by Eq. (1) versus |*B*/*J*| for a mixed-valent Fe^2+^(*S* = 2)-Fe^3+^(*S* = 5/2) compound, for *J* > 0 and *J* < 0. The expansion of the energy levels for *J* < 0 (right) exhibits intermediate spin states in the region 3 ≤ |*B*/*J*| ≤ 9. **b**, General chemical representation of the anion [L_2_Fe_2_Q_2_]^−^ of complexes **1**–**3**. The red arrows highlight the PKS vibration (relative nuclear displacement). **c**–**e**, Adiabatic ground- and excited-state potential surfaces of various examples of the Heisenberg double-exchange formalism expanded to include vibronic coupling as described by PKS (Eq. (2)), where a partially delocalized *S* = 1/2 ground spin state (**c**), a fully delocalized *S* = 9/2 ground spin state (**d**) and intermediate spin states (**e**) may be stabilized.

Although the electronic structures of mixed-valent complexes are typically dominated by Heisenberg exchange coupling, *J*, systems displaying substantial double-exchange, *B*, have attracted increasing attention (refs. ^9,14–16^). The energy levels for a (locally high-spin) *d*^5^-*d*^6^ mixed-valent pair, such as a [Fe_2_Q_2_]^+^ cluster, are plotted in the energy correlation diagram of Fig. 1a. In the antiferromagnetically coupled case, *J* < 0, the two documented spin states for Fe^II^-Fe^III^ dimers of *S* = 1/2 and 9/2 are stabilized at the extremities of the plot, where *J* and *B* dominate the magnetic coupling interaction of the unpaired electron, respectively. For ratios of |*B*/*J*| in the range 3 ≤ |*B*/*J*| ≤ 9, discrete intermediate spin states *S* = 3/2, 5/2 and 7/2 are predicted. The Heisenberg double-exchange model has also been expanded for larger clusters, such as [Fe_4_S_4_]^+^ centres, to formally explain the various intermediate spin states observed in these cubane systems^12,13,17–20^. Although the stabilization of intermediate spin states for dimers has long been predicted, to date only the extrema spin states, *S* = 1/2 and *S* = *n*/2, have been reported^5,7,9,16^.

The inclusion of vibronic coupling, as first introduced by Piepho, Krausz and Schatz (PKS)^21^, further modulates the spin levels, favouring electronic localization of the unpaired electron and resulting in destabilization of the intermediate spin states, making the isolation of intermediate spin state dimers all the more challenging. Incorporation of the PKS vibronic coupling into the Heisenberg double-exchange model yields final energy states determined by the following equation:

2 $$\begin{array}{l}{{{E}}}_ {\pm} = - {{{JS}}}\left( {{{{S}}} + 1} \right) + \frac{1}{2}\left( {\frac{{\lambda ^2}}{{{{{k}}}_{\_}}}} \right){{{x}}}_{\_}^2\\\qquad\ \pm \left[ {\frac{1}{2}\left( {\frac{{\lambda ^2}}{{{{{k}}}{\_}}}} \right)^2{{{x}}}_{\_}^2 + {{{B}}}^2\left( {{{{S}}} + \frac{1}{2}} \right)^2} \right]^{\frac{1}{2}}\end{array}$$

where *λ*^2^/*k*_–_ is the vibronic coupling term over vibrational coordinate *x*_−_. The major localizing vibronic coupling mode previously identified in mixed-valent dimers is the PKS vibration^22^, an out-of-phase breathing mode that enhances electronic localization through geometric desymmetrization (Fig. 1b). For typical [Fe_2_S_2_]^+^ clusters, the inclusion of vibronic coupling results in a double-well *S* = 1/2 ground spin state (Fig. 1c), demonstrating partial electron delocalization. Even though double exchange is estimated to dominate over Heisenberg exchange in various [Fe_2_S_2_]^+^ clusters^5,23^, the majority still exhibit *S* = 1/2 ground states. Instead, localizing forces such as hydrogen-bonding interactions and vibronic coupling counter the delocalizing effect of double exchange to arrive at the experimentally observed partially delocalized *S* = 1/2 ground states. These partially delocalized mixed-valent dimers fit within class II of the Robin–Day system, where a low-energy activation barrier allows for interconversion of the dimer’s valency (in contrast to fully localized class I systems that exhibit no interconversion)^24^. Only a few examples of mutant [Fe_2_S_2_]^+^ proteins and synthetic complexes have been found to exhibit the fully delocalized ‘antiferromagnetically’ coupled *S* = 9/2 spin state (*J* < 0 and |*B*/*J*| > 9), fitting into class III of the Robin–Day classification system^3–10^. In these cases of very large |*B*/*J*|, modestly large vibronic couplings, such as that observed in the [Fe_2_(OH)_3_]^3+^ complex^5^, still do not overcome the Heisenberg double-exchange interaction, resulting in a single-well *S* = 9/2 ground spin state with complete electron delocalization (Fig. 1d), These few examples demonstrate that under certain conditions the localizing effects of vibronic coupling and other trapping forces can be surmounted^4,5,25^, further suggesting that intermediate spin states may be achievable within the vibronic coupling-extended formalism of Heisenberg double exchange (Fig. 1e).

Previous studies have highlighted that moderate-to-strong vibronic coupling results in a vanishing of the intermediate spin states for a given spin ladder^3,26^, and consequently minimization of vibronic coupling is crucial for stabilization of intermediate spin states. As biological [Fe_2_S_2_]^+^ mutants and synthetic [Fe_2_OR_2/3_]^3+/2+^ complexes have overcome vibronic coupling to access *S* = 9/2 spin states, we have explored the substitution of heavier chalcogenide bridges in mixed-valent diiron complexes as a means of accessing intermediate spin states. Through substitution of the bridging ligands from S to Se to Te, we anticipated that the PKS vibration, and thus overall vibronic coupling, would diminish with the increasing mass of the chalcogenide, potentially allowing for stabilization of previously unobserved spin states in mixed-valent dimers.

## Results and discussion

**Fig. 2:**
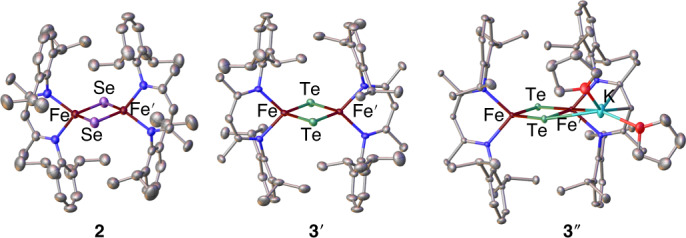
Single-crystal X-ray diffraction structures of mixed-valent diiron dichalcogenide complexes. The structures of [L_2_Fe_2_Se_2_][K(THF)_6_]·2THF (**2**, left; Supplementary Fig. 7), [L_2_Fe_2_Te_2_][K(THF)_6_]·2THF (**3**′, centre; Supplementary Fig. 8) and L_2_Fe_2_Te_2_K(THF)_2_ (**3**″, right; Supplementary Fig. 9) drawn with 50% probability thermal ellipsoids. Nitrogen atoms are shown in blue, oxygen atoms in red and carbon atoms in grey. Hydrogen atoms, solvent molecules and outer-sphere potassium cations are omitted for clarity. Only one crystallographically distinct anion is shown for **3**′.

We have synthesized and structurally characterized (Fig. 2) a series of [L_2_Fe_2_Q_2_]^−^ dimers (with tetrahydrofuran (THF)-solvated K^+^ counter ions) supported by a β-diketiminate ligand (L^−^), where the bridging µ-Q^2–^ ligands are S^2–^ (**1**)^27^, Se^2–^ (**2**) and Te^2–^ (**3**). To the best of our knowledge, compound **3** is the first reported synthesis of an [Fe_2_Te_2_]^+^ complex. Single-crystal X-ray diffraction studies confirmed the formal oxidation state [Fe_2_Q_2_]^+^ and revealed metrical parameters around the Fe centres consistent with high-spin pseudo-tetrahedral ferric/ferrous ions for complexes **1**–**3** (Supplementary Tables 1 and 2, and Supplementary Figs. 5–9). It is notable that complex **3** crystallizes in two forms, **3**′ and **3**″, with an outer-sphere and inner-sphere potassium counter ion, respectively. In general, the anion [L_2_Fe_2_Te_2_]^−^ will be referred to as **3**, while specific crystalline forms will be referred to as **3**′ and **3**″.

Successive chalcogenide substitution in the [Fe_2_Q_2_]^+^ core elongates the Fe–Fe distance by around 0.15 Å per substitution, with the Fe–Fe distance increasing from 2.807(1) Å in the disulfide complex **1** to 3.172(1) Å in the ditelluride complex **3** (average of the three crystallographically unique Fe–Fe distances in **3**′ and **3**″), consistent with the larger atomic radii of Se and Te. Of note, **1** and **2** exhibit longer Fe–Fe distances than previously reported [Fe_2_Q_2_]^+^ complexes containing pseudo-tetrahedral iron centres^28–30^, which we attribute to the steric repulsion generated by the bulky isopropyl groups of the flanking β-diketiminate ligands.

**Fig. 3:**
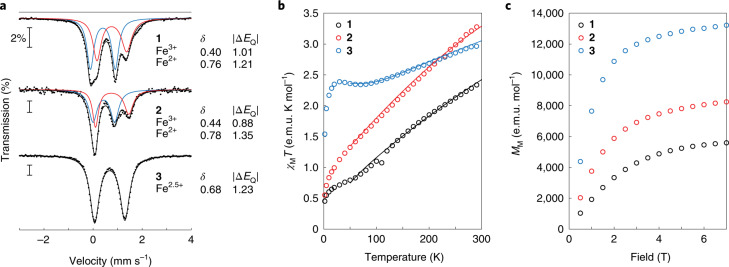
Zero-field Mössbauer spectroscopy and magnetization measurements. **a**, ^57^Fe Mössbauer spectra of **1**–**3** collected at 80 K and 0 T (dots), and fits evidencing partially localized (class II Robin–Day classification) ‘Fe^2+^’ (red) and ‘Fe^3+^’ (blue) sites for **1** and **2** (sum in black). The Mössbauer spectrum of **3** is fit with a single ‘Fe^2.5+^’ site (black) consistent with a fully delocalized (class III) mixed-valent dimer. Individual isomer shifts, *δ*, and quadrupole splitting, |Δ*E*_Q_|, are detailed to the right of the spectra in units of mm s^−1^. **b**, Variable-temperature SQUID magnetic susceptibility measurements of solid samples of **1**–**3** exhibit increasing magnetic responses at low temperatures across the series. The higher-temperature (>50 K) measurements are fit to the solved Bleaney–Bowers equation for a *d*^5^-*d*^6^ mixed-valent centre with the incorporation of double-exchange coupling (Supplementary Fig. 17). The fits shown are solutions with |*B*/*J*| ratios of 2.0 ± 0.2, 2.9 ± 0.2 and 3.7 ± 0.1 for **1**, **2** and **3**, respectively (e.m.u., electromagnetic unit.). **c**, SQUID saturation magnetization measurements of solid samples of **1**–**3** recorded at 2 K. The calculated magnetic moments at saturation are 1.6 ± 0.1 *μ*_B_, 2.5 ± 0.1 *μ*_B_ and 4.3 ± 0.1 *μ*_B_ per molecule for complexes **1**, **2** and **3**, respectively (Supplementary Fig. 21).

The zero-field ^57^Fe Mössbauer spectra for crystalline complexes **1** and **2** recorded at 80 K (Fig. 3a and Supplementary Table 3) show that the isolated bulk material is consistent with an assignment of antiferromagnetically coupled mixed-valent centres^31,32^. The spectra of **1** and **2** are well fit with two overlapping quadrupole doublets in 1:1 ratios, formally representing the locally high-spin Fe^2+^ and Fe^3+^ ions. The Mössbauer spectrum of **3** exhibits a single quadrupole doublet response at 80 K at an isomer shift intermediate to the individual ‘Fe^2+^’ and ‘Fe^3+^’ isomer shifts of **1** and **2**, suggesting **3** is fully valence-delocalized, ‘Fe_2_^2.5+^-Fe_2_^2.5+^’. Based on these results, as well as variable-temperature studies (Supplementary Figs. 13 and 14), complexes **1** and **2** are assigned as partially delocalized Robin–Day class II, whereas complex **3** is assigned as fully delocalized class III (Supplementary Fig. 15).

Superconducting quantum interference device (SQUID) magnetization measurements of **1**–**3** (Fig. 3b**)** revealed increasing molar magnetic susceptibility, *χ*_M_*T*, responses at low temperatures (<50 K) across the series, with the *χ*_M_*T* responses of **1** and **3** suggestive of *S* = 1/2 and *S* = 3/2 ground states, respectively, whereas **2** exhibited an intermediate response. Without magnetic impurities (Supplementary Fig. 16), the positive pseudo-linear *χ*_M_*T* temperature dependence observed above 50 K for **1**–**3** fits well the Bleaney–Bowers equation^33^ for magnetic susceptibility with the inclusion of double exchange using |*B*/*J*| ratios of 2.0 ± 0.2, 2.9 ± 0.2 and 3.7 ± 0.1 for **1**, **2** and **3**, respectively. These fitted ratios of |*B*/*J*| place **1** and **3** comfortably within the *S* = 1/2 and 3/2 regions, respectively, of the Heisenberg double-exchange spin ladder (Fig. 1), whereas the fitted |*B*/*J*| ratio for **2** is near the crossing point of *S* = 1/2 and *S* = 3/2. The Heisenberg double-exchange model allows for robust fitting of the |*B*/*J*| ratio only for the higher-temperature magnetic susceptibility data (Supplementary Table 4, Supplementary Figs. 17–20 and Supplementary ‘Discussion of Limitations of the HDE Model to Magnetic Data Fitting’), as previously demonstrated in the double-exchange dominated vanadium dimer^3^ and other higher-nuclearity FeS clusters^19,34,35^. The ground spin states for complexes **1**–**3** were additionally probed through saturation magnetization measurements recorded at 2 K (Fig. 3c), demonstrating increasing molar magnetization, *M*_M_, across the series with **1** < **2** < **3**. Accounting for the random orientation of crystallites within the powder, the magnetic moments along the main magnetization axis at saturation were calculated to be 1.6 ± 0.1 *μ*_B_, 2.5 ± 0.1 *μ*_B_ and 4.3 ± 0.1 *μ*_B_ per molecule for complexes **1**, **2** and **3** (Supplementary Fig. 21). These magnetic moments are consistent with *S* = 1/2 and 3/2 ground states for **1** and **3**, respectively, whereas the intermediate magnetic moment of **2** compared with **1** and **3** suggests a mixture of *S* = 1/2 and 3/2 ground states within the sample.

**Fig. 4:**
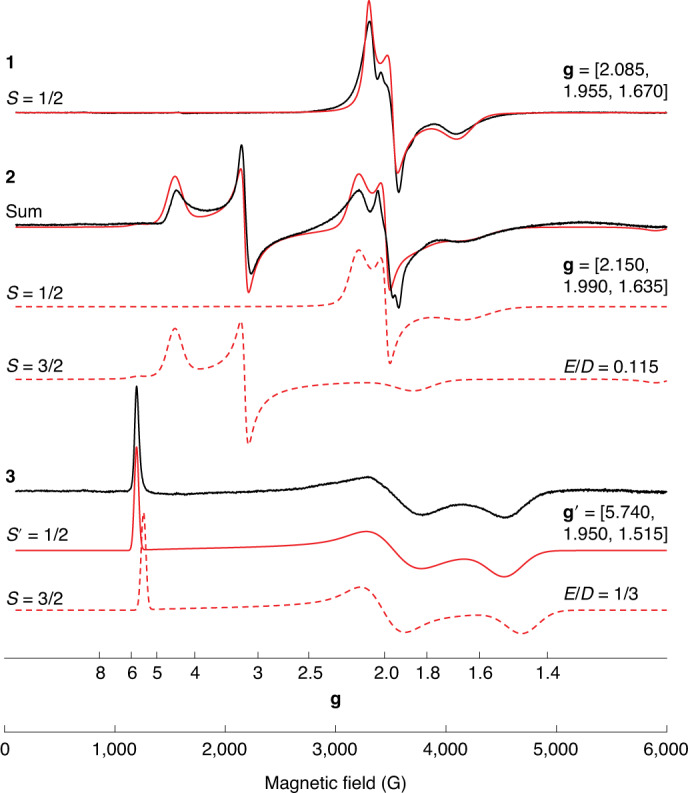
Low-temperature X-band EPR spectroscopy directly evidences *S* = 1/2 and *S* = 3/2 spin states of mixed-valent [Fe_2_Q_2_]^+^ complexes. Continuous-wave X-band (~9.63 GHz) EPR spectra of **1**–**3** as frozen solutions in acetone at 3.6–8 K displayed as collected under non-saturating microwave power conditions (black lines). Simulations are shown as red lines. Complex **1** is simulated as an *S* = 1/2 spin system with **g** = [*g*_1_, *g*_2_, *g*_3_] = [2.085, 1.955, 1.670], 90 G full-width half-maximum (fwhm) Lorentzian line broadening and an additional 0.1 fhwm Gaussian *g*_3_ strain distribution. The simulated spectrum of **2** is composed of equal-summed components of *S* = 1/2 and *S* = 3/2 signals (±5% weight). The *S* = 1/2 simulation parameters are **g** = [2.150, 1.990 1.635], 70 G fwhm Lorentzian line broadening and *g* strain (Gaussian distribution) of [0.09, 0, 0.17]. The *S* = 3/2 component parameters are *g*_iso_ = 1.925, |*D*| ≫ *hν*, *E*/*D* = 0.115 and *g* strain = [0, 0.15, 0.18]. The *S* = 3/2 EPR spectrum of **3** is simulated with an *S*′ = 1/2 spin representation with parameters **g**′ = [5.740, 1.950, 1.515], 45 G fwhm line broadening and *g* strain = [0, 0.25, 0.12]. For the *S* = 3/2 simulation of **3**, a *g*_iso_ value is not well estimated and *g*_e_ is used. The EPR spectrum of **3** is also reproduced well by an *S* = 3/2 spin system with parameters |*D*| ≫ *hν*, |*E*/*D*| = 1/3 and *D* strain = 0.1*D*.

Low-temperature continuous-wave X-band (~9.63 GHz) electron paramagnetic resonance (EPR) experiments performed under non-saturating microwave conditions yielded dramatically different spectra for complexes **1–3** (Fig. 4 and Supplementary Figs. 22 and 23). The solution spectrum of **1** exhibits a rhombic *S* = 1/2 signal with a *g*_iso_ value of ~1.90 (*g*_iso_ = (*g*_1_ + *g*_2_ + *g*_3_)/3), consistent with previously studied biological and synthetic [Fe_2_S_2_]^+^ clusters supported by nitrogen ligands^36–38^. The variable-temperature EPR response of **1** was observed to decrease in intensity with rising temperature until ~60 K, where the signal was nearly entirely lost (Supplementary Fig. 24). Ferredoxin [Fe_2_S_2_]^+^ clusters and synthetic analogues typically exhibit EPR signals at temperatures >80 K (ref. ^39^), indicating unusually faster spin relaxation for **1**.

Selenium substitution substantially changes the electronic structure of **2**, relative to **1**, as seen by the appearance of new low-field features at *g* = 4.38 and 3.16 (Fig. 4). The EPR spectrum of **2** comprises two equally weighted EPR components, an *S* = 1/2 signal similar to **1** (albeit with a slightly larger *g*_iso_ value of 1.92 and increased anisotropic character, Δ = *g*_1_ – *g*_3_) and a new *S* = 3/2 signal. It is noted that both **1** and **2** exhibit a minor (<1%) *S* = 1/2 impurity that is more apparent and sharper than the corresponding [Fe_2_Q_2_]^+^ EPR signal at higher temperature (Supplementary Fig. 25). The *S* = 3/2 signal of **2** is well reproduced by simulation using the determined *g*_iso_ value of the *S* = 1/2 signal of **2**, and a large zero field splitting, *D*, greater than the microwave incident energy (|*D*| ≫ *hν* ≈ 0.3 cm^−1^), and a rhombic zero-field splitting tensor represented by the ratio *E*/*D* = 0.115 (Fig. 4). The observed and predicted *g* values for the *S* = 3/2 signal of **2** belong to the *M*_s_ = ±1/2 spin manifold with a positive *D* value^12^. The assignment of an *S* = 3/2 spin state in **2** is further supported by spin nutation measurements (Supplementary Figs. 26 and 27). Between 3.6 and 8.0 K, the variable-temperature EPR spectra of **2** reveal no changes in the line shapes, positions or relative intensities of the *S* = 1/2 and 3/2 signals (Supplementary Fig. 28). The independent nature of the two signals indicates that the spectra are the result of an approximately equal physical mixture of *S* = 1/2 and *S* = 3/2 ‘spin isomers’ in the frozen solution^40^. Above 8 K, the *S* = 3/2 signal of **2** broadens due to a larger Lorentzian linewidth contribution from the increased population of the higher-energy *M*_s_ = ±3/2 doublet of the zero-field split spin manifold. Lastly, the electron nuclear double resonance (ENDOR) spectra of the *S* = 1/2 signals of **1** and **2** (Supplementary Fig. 29) reveal distinct ^57^Fe^3+^ isotropic hyperfine couplings (~48 and 45 MHz, respectively), with the isotropic hyperfine coupling slightly reduced in **2** due to the increased covalency of the Se ligands and/or increased delocalization of the unpaired electron (also suggested by the Mössbauer experiments). Attempts to collect ^57^Fe ENDOR spectra of the *S* = 3/2 signals were unsuccessful, possibly due to the faster relaxation times of the high-spin system.

Tellurium substitution further changes the electronic structure relative **1**, with the EPR spectrum of **3** exhibiting a single response (consistent with complete potassium solvation) with a sharp low-field transition at *g* = 5.740 and broader transitions at higher field (Fig. 4), characteristic of an *S* = 3/2 signal with large zero-field splitting and a completely rhombic zero-field splitting tensor represented by |*E*/*D*| = 1/3. Due to the complete rhombicity of **3**, the sign of *D* may not be determined from inspection of the *g* values^12^. The spectrum of **3** may be reproduced by simulation using a fictitious *S*′ = 1/2 spin representation^41^, to refine the effective *g* values of **g**′ = [5.740, 1.950, 1.515]. Employing an *S* = 3/2 spin Hamiltonian with large zero-field splitting and |*E*/*D*| = 1/3 also reproduces the observed experiment well, but a slight disagreement of the low-field transition is observed due to the assumption that **g** = *g*_e_. Once again, the EPR spectra of **3** are broadened at higher temperatures due to mixing of the other zero-field splitting doublet (Supplementary Fig. 30). From the estimated Lorentzian linewidth component observed in **3**, the zero-field splitting is estimated to be a maximum of *D* = 15 cm^−1^ (Supplementary Fig. 31). As previous [Fe_2_Q_2_]^+^ systems have only exhibited *S* = 1/2 or 9/2 spin states, these spectra represent the first observations of *S* = 3/2 signals by EPR spectroscopy for such clusters. These results are also consistent with the interpretation of the SQUID data and fitted |*B*/*J*| ratios, with **1** exhibiting an *S* = 1/2 spin state, **2** a mixture of *S* = 1/2 and 3/2, and **3** exhibiting only *S* = 3/2.

In the presence of applied external magnetic fields, complex **1** exhibits magnetically split Mössbauer spectra (Supplementary Fig. 32) qualitatively similar to a previously reported synthetic [Fe_2_S_2_]^+^ complex^6^ and can be simulated using a near-isotropic hyperfine tensor for the ‘Fe^3+^’ site of **A**(Fe^3+^) = [−35 T, −31 T, −39 T] and an anisotropic hyperfine tensor for the ‘Fe^2+^’ site of **A**(Fe^2+^) = [−5 T, 16 T, 13 T] (Supplementary Table 5). Complex **2** exhibits magnetic Mössbauer spectra (Supplementary Fig. 33) that are more collapsed compared with those of **1**, behaviour previously observed in *S* = 3/2 and spin-admixed [Fe_4_Q_4_]^3+^ cubanes (Q = S, Se)^40^. The magnetic Mössbauer spectra of **2** can be satisfactorily simulated using a 70:30 mixture of *S* = 1/2 and *S* = 3/2 spins, with the *S* = 1/2 subspectrum similar to the simulation for **1** (albeit with a slightly smaller isotropic ferric hyperfine coupling of −30 T (~41.4 MHz), consistent with the ENDOR results) and the *S* = 3/2 subspectrum similar to the simulation for **3** (vide infra), using **g** = [4.38, 3.16, 1.96], |*E*/*D*| = 0.12, *D* = +11 cm^−1^ and an isotropic ^57^Fe hyperfine tensor of −10 T. The magnetic Mössbauer spectrum of complex **3** exhibits distinct splitting (Supplementary Fig. 34) that can be readily simulated for an *S* = 3/2 centre using **g** = [5.740, 1.950, 1.515], |*E*/*D*| = 0.33, *D* = +11 cm^−1^ and an isotropic ^57^Fe hyperfine tensor of −10 T. The fitted ^57^Fe hyperfine couplings of the *S* = 3/2 spins in **2** and **3** are half that typically observed for FeS clusters, indicating substantial delocalization similar to the mixed-valence pairs in FeS cubane clusters with intermediate ground states^40,42^. These results are fully consistent with the EPR and SQUID studies, and confirm the assignment of **3** unambiguously as having *S* = 3/2 intermediate spin.

In addition to the Mössbauer isomer shifts and single-crystal X-ray diffraction structural parameters supporting locally high-spin Fe centres (vide supra), the local electronic structures of complexes **1**–**3** were further probed through Fe Kβ X-ray emission spectroscopy (Fe 3*p*→1*s*), which reports on spin state through exchange coupling of the 3*p*-3*d* manifolds^43^. The Fe Kβ X-ray emission spectra of **1–3** (Supplementary Fig. 35) are indicative of locally high-spin Fe^2+/3+^ centres, precluding the possibility of locally intermediate- or low-spin Fe centres to achieve the observed intermediate spin states^44^ and instead support the double-exchange delocalization mechanism.

Density functional theory (DFT) calculations of both high-spin (*S* = 9/2) and broken-symmetry (*S* = 1/2) solutions were performed for complexes **1**–**3**. Although DFT and other state-of-the-art wavefunction-based methods^45^ are incapable of accurately predicting the intermediate *S* = 3/2 ground state of complexes **2** and **3**, the broken-symmetry *S* = 1/2 solutions reproduce well the structural parameters and Mössbauer trends across the series (Supplementary Tables 6 and 7) and may offer insight into the influence of the chalcogenide on the overall electronic structure of these clusters. The calculations (Supplementary Table 8) suggests a trend of modestly increasing (more positive) *J* values (that is, weaker antiferromagnetic coupling) with heavier chalcogenide substitution, both for the diferric and mixed-valent series, in agreement with previous wavefunction-based calculations^45^. Analysis of the Mulliken spin populations (Supplementary Table 9) reveals greater spin parity on the Fe centres (balanced by increased majority spin density on the chalcogenide bridges) with heavier chalcogenide incorporation, consistent with increased delocalization of the itinerant electron onto the heavier chalcogenide bridges.

Furthermore, frequency calculations (Supplementary Table 10) demonstrate that heavier chalcogenide substitution results in shifting of the PKS vibration to lower energy from 295 cm^−1^ in **1** to 160 cm^−1^ in **2** and 141 cm^−1^ in **3**. In addition to the reduction in vibrational frequency of the PKS mode, the calculations reveal that the magnitude of the bridging chalcogenide displacements also decreases in the heavier Se and Te complexes compared with in S (Supplementary Fig. 36), and thus substitution of the sulfide bridge by heavier congeners reduces the charge localizing effect of the PKS vibration in **2** and **3** by limiting the geometric desymmetrization induced by the PKS mode. This effect was observed not only to be a function of the chalcogenide, but also of the Fe–Fe distance and supporting ligand substitution, suggesting the steric bulk of the β-diketiminate ligand in the present series also facilitates minimization of the PKS localization effect. Analysis of the vibronic coupling term (*λ*^2^/*k*_, Supplementary Table 11) derived from the calculated PKS vibrations shows a near-halving from **1** to **2** (2,200 to 1400 cm^−1^), and only a modest increase from **2** to **3** (1,400 to 1,650 cm^−1^). (Note: these values are likely overestimates as similar displacements were used for all three compounds for simplicity, while the PKS visualizations reveal clearly smaller displacements for the heavier chalcogenides.) Combining these values with estimates for *B* and *J* based on the DFT calculations and SQUID data fittings (Supplementary Table 11), ground and excited spin state adiabatic potential surfaces could be generated for complexes **1**–**3** (Supplementary Fig. 37). These potential surfaces reveal the experimentally observed *S* = 1/2 ground spin state for **1**, a more condensed spin ladder for **2** with *S* = 1/2, 3/2 and 5/2 all close in energy, and the well-isolated *S* = 3/2 ground spin state for **3**. These results illustrate the competing effects of Heisenberg exchange, double-exchange and vibronic coupling across the series and underscore the delicate balance achieved through heavier chalcogenide substitution in the [Fe_2_Q_2_]^+^ core.

## Conclusion

In summary, we have reported here the synthesis of transition metal dimer complexes that stabilize intermediate spin ground states. Through a combination of magnetic susceptibility, Mössbauer and EPR measurements, as well as computational analysis, we have shown that Se and Te incorporation in the diiron dichalcogenide core results in weakening antiferromagnetic coupling between the metal centres, increased electronic delocalization and decreased vibronic coupling, allowing for the stabilization of the intermediate spin states. Importantly, this diiron dichalcogenide series may represent a platform for deepening our understanding of electronic structure in synthetic and biological systems.

## Methods

### General considerations

Unless indicated otherwise, all manipulations were performed using oven-dried glassware in an M-Braun nitrogen-atmosphere glove box or on a Schlenk line using standard Schlenk techniques. Molecular sieves were activated by heating at 200 °C for 48 h under high vacuum. THF, toluene, diethyl ether, hexanes and pentane were purchased anhydrous from Sigma, further dried over sodium/benzophenone ketyl, vacuum-transferred before use and stored over 4 Å molecular sieves. KC_8_, C_10_H_8_ and *n*-BuLi (2.5 M in hexanes) were purchased from Sigma and used as received. ^57^Fe metal was purchased from EurIsotop (Cambridge Isotope Laboratories) and used as received. SePMe_3_, TePCy_3_ (Cy = cyclohexyl), LH, ^57^FeCl_2_, LFe(PhMe), L_2_Fe_2_S_2_ (**1**^**ox**^) and [K(THF)_6_][L_2_Fe_2_S_2_]·2THF (**1**) were prepared as previously reported^27,46–50^.

### Synthesis of L_2_Fe_2_Se_2_ (2^ox^)

A solution of SePMe_3_ (0.1413 g, 0.911 mmol) in PhMe (4 ml) was added to a red-brown solution of LFe(PhMe) (0.5044 g, 0.892 mmol) in PhMe (3 ml). Upon addition, the solution lost its red hue, turning dark brown with concomitant production of dark-green crystals. After 1 h at room temperature, the crystals were collected on a glass frit and washed with THF until the filtrate ran clear (~4 ml). The dark-green crystals were dried under vacuum to afford 0.3218 g (65% yield) of the desired complex. This compound demonstrates very poor solubility in all common laboratory solvents tested and was used without further purification. Single crystals suitable for X-ray diffraction (XRD) were prepared by combining filtered solutions of LFe(PhMe) (0.0221 g) and SePMe_3_ (0.0057 g) in THF (1 ml each) in a 4 mL vial. The vial was capped and inverted several times and left to sit at room temperature for 24 h. IR, *v* (cm^−1^): 458(w), 528(w), 565(w), 626(w), 640(w), 695 (s), 729(w), 759(s), 773(m), 795(s), 852(m), 930(m), 1,022(m), 1,057(w), 1,098(m), 1,175(m), 1,253(m), 1,275(w), 1,315(s), 1,363(s), 1,380(s), 1,434(m), 1,458(m), 1,518(m), 1,530(m), 2,863(w), 2,922(w), 2,955(m), 3,055(w). Elemental analysis calculated for [L_2_Fe_2_Se_2_], C_58_H_82_Fe_2_N_4_Se_2_: C, 63.05; H, 7.48; N, 5.07. Found: C, 63.89; H, 7.58; N, 4.82.

### Synthesis of [K(THF)_6_][L_2_Fe_2_Se_2_]·2THF (2)

Solid KC_8_ (0.0263 g, 0.195 mmol) was added to a stirred suspension of the crude diferric complex **2**^**ox**^ (0.2002 g, 0.181 mmol) in THF (3 ml). The reaction mixture was stirred for 1 h, at which point the dark-green precipitate had been consumed to afford a dark-brown solution. The resultant mixture was filtered through a glass microfibre filter pad to remove graphite, rinsing with additional THF (~3 ml). The dark-brown filtrate was then concentrated under vacuum. The residue was triturated with Et_2_O (3 ml) and filtered through a glass microfibre filter pad. The resulting black microcrystalline solid was washed with additional Et_2_O until the filtrate ran clear (~4 ml). The black solid was then resolubilized in THF (~6 ml), filtered through the glass microfibre pad, further concentrated to half-volume under vacuum and stored at –35 °C to afford 0.1291 g (41% yield) of black crystals (suitable for XRD). UV-vis (THF, 21 °C), *λ*_max_ (nm), *ε* (M^−1^ cm^−1^): 390, 1.8 × 10^4^; 430, 1.33 × 10^4^; 510, 7.4 × 10^3^; 555, 6.6 × 10^3^; 645, 4.1 × 10^3^; 720, 2.6 × 10^3^. IR, *v* (cm^−1^): 441(w), 459(w), 525(w), 628(w), 638(w), 712(w), 759(s), 793(s), 846(w), 930(w), 1,020(w), 1,059(w), 1,101(w), 1,175(m), 1,256(m), 1,316(s), 1,364(s), 1,382(s), 1,434(m), 1,460(m), 1,512(m), 1,523(m), 2,865(w), 2,925(w), 2,960(m), 3,059(w). Elemental analysis calculated for [K(THF)_6_][L_2_Fe_2_Se_2_]·2THF, C_90_H_146_Fe_2_KN_4_O_8_Se_2_: C, 62.82; H, 8.55; N, 3.26. Found: C, 62.06; H, 8.29; N, 3.63.

### Synthesis of L_2_Fe_2_Te_2_ (3^ox^)

A filtered solution (glass microfibre) of TePCy_3_ (0.3758 g, 0.921 mmol) in PhMe (3 ml) was added to a filtered solution of LFe(PhMe) (0.5099 g, 0.901 mmol) in PhMe (3 ml) in a 20-ml scintillation vial. The vial was capped and the resulting red-brown mixture was inverted in the vial several times, then left to sit at room temperature for 24 h, during which dark crystals formed. The crystals were collected on a glass frit and washed with Et_2_O (3 × 10 ml). The collected black crystals were dried under vacuum to afford 0.3815 g (64%) of the desired complex as the toluene solvate. This compound demonstrates very poor solubility in all common laboratory solvents tested and was used without further purification. Single crystals suitable for XRD were prepared by layering a filtered solution of TePCy_3_ (0.0149 g) in PhMe (1.5 ml) on a filtered solution of LFe(PhMe) (0.0195 g) in PhMe (1.5 ml) in a 4 mL vial. The vial was capped and left to sit at room temperature overnight, resulting in the growth of black crystals. IR, *v* (cm^−1^): 410(w), 438(w), 454(w) 528(w), 565(w), 600(w), 628(w), 640(w), 690(s), 715(w), 730(w), 756(s), 765(m), 792(s), 855(m), 900(w), 930(m), 1,022(m), 1,055(w), 1,100(m), 1,175(m), 1,262(m), 1,315(s), 1,361(s), 1,384(s), 1,437(m), 1,451(m), 1,462(m), 1,489(m), 1,518(s), 1,600(w), 2,864(w), 2,924(w), 2,960(m), 3,022(w), 3,062(w). Elemental analysis calculated for [L_2_Fe_2_Te_2_·3PhMe], C_79_H_106_Fe_2_N_4_Te_2_: C, 64.17; H, 7.23; N, 3.79. Found: C, 64.28; H, 7.12; N, 4.09.

### Synthesis of L_2_Fe_2_Te_2_K(THF)_2_ and [K(THF)_6_][L_2_Fe_2_Te_2_]·2THF (3)

A solution of KC_10_H_8_ was generated by stirring KC_8_ (0.0415 g, 0.307 mmol) with naphthalene (0.0366 g, 0.286 mmol) in THF (3 ml). After 30 min, the dark-green KC_10_H_8_ solution was added dropwise to a stirring suspension of **3**^**ox**^ (0.3017 g, 0.250 mmol) in THF (5 ml). Upon complete addition, the **3**^**ox**^ had solubilized to give a dark-red solution. The reaction mixture was stirred for 1 h and then all volatiles were removed under vacuum. The resulting dark residue was triturated with Et_2_O (5 ml) and filtered through a glass microfibre filter pad. The resulting black-red solid was washed with additional Et_2_O until the filtrate ran clear (~5 ml). The black-red solid was then resolubilized in THF (~5 ml), filtered through the glass microfibre pad, further concentrated to half-volume under vacuum and stored at –35 °C to afford 0.1851 g (46% yield) of black-red crystals (suitable for XRD). Single-crystal XRD analysis revealed two different structures, **3**′ and **3**″, in roughly a 1:1 ratio, as suggested by powder XRD (Supplementary Fig. 11). UV-vis (THF, 21 °C), *λ*_max_ (nm), *ε* (M^−1^ cm^−1^): 320, 4.7 × 10^4^; 410, 1.25 × 10^4^; 510, 7.3 × 10^3^; 655, 2.95 × 10^3^; 785, 3.1 × 10^3^; 845, 2.8 × 10^3^. IR, *v* (cm^−1^): 431(w), 460(w), 524(w), 628(w), 640(w), 713(w), 758(s), 792(s), 845(w), 895(w), 930(m), 1,022(m), 1,052(m), 1,100(m), 1,175(m), 1,258(m), 1,316(s), 1,361(s), 1,382(s), 1,435(s), 1,460(m), 1,523(m), 2,865(w), 2,926(w), 2,956(m). Elemental analysis calculated for [L_2_Fe_2_Te_2_K(THF)_5_], C_78_H_122_Fe_2_KN_4_O_5_Te_2_: C, 58.49; H, 7.68; N, 3.50. Found: C, 58.21; H, 7.66; N, 3.59.

### Synthesis of [L_2_^57^Fe_2_(N_2_)]

This compound was prepared using a modification of the literature procedure^51^. *n*-BuLi (2.5 M, 0.50 ml, 0.125 mmol) was added dropwise to a stirred solution of LH (0.5166 g, 1.23 mmol) in THF (5 ml), generating a pale-yellow mixture. After stirring for 20 min, ^57^FeCl_2_ (0.1597 g, 1.25 mmol) was added to the LiL solution with the aid of THF (~2 ml). The resultant yellow-brown mixture was stirred at room temperature for 1 h. Solid KC_8_ (0.0177 g, 1.31 mmol) was then added to the reaction mixture, resulting in a dark-brown suspension. After stirring for 1 h and visually confirming the consumption of KC_8_, the reaction mixture was filtered through a glass microfibre filter pad to remove graphite, rinsing with additional THF (3 ml). The brown filtrate was then concentrated under vacuum. The resulting residue was extracted with pentane (~5 ml) and filtered through a glass microfibre filter pad. The resulting red filtrate was concentrated to half-volume and stored at –35 °C to afford 0.2786 g (46%) of the desired complex as dark-red crystals (confirmed by ^1^H NMR).

### Synthesis of L_2_^57^Fe_2_S

This compound was prepared using a modification of the literature procedure^51^. A solution of L_2_^57^Fe_2_(N_2_) (0.1799 g, 0.185 mmol) in Et_2_O (3 ml) was frozen in a liquid nitrogen-cooled cold well. Separately, a solution of SPMe_3_ (0.0204 g, 0.189 mmol) in Et_2_O (3 ml) was also frozen in a liquid nitrogen-cooled cold well. Upon thawing, the SPMe_3_ solution was added to the L_2_^57^Fe_2_(N_2_) solution and the combined mixture stirred while allowing to warm to room temperature. After stirring for 1 h at room temperature, the volatiles were removed under vacuum. The residue was then extracted with pentane (5 ml) and filtered through a glass microfibre filter pad. The red filtrate was concentrated to half-volume and stored at –35 °C to afford 0.1254 g (69%) of the desired complex as dark-red crystals (confirmed by ^1^H NMR).

### Synthesis of [K(THF)_6_][L_2_^57^Fe_2_S_2_] (^57^1)

This compound was prepared analogously to the non-isotopically labelled **1** as previously reported with L_2_^57^Fe_2_S (0.1254 g, 0.128 mmol) and SSbPh_3_ (0.0497 g, 0.129 mmol) to afford 0.0293 g (23%) of the crude isotopically labelled diferric ^**57**^**1**^**ox**^ (L_2_^57^Fe_2_S_2_)^27^. Reduction was accomplished with KC_8_ (0.0054 g, 0.040 mmol) and C_10_H_8_ (0.0048 g, 0.037 mmol) to afford 0.0122 g of ^**57**^**1** as dark-red crystals in 6% overall yield.

### Synthesis of [K(THF)_6_][L_2_^57^Fe_2_Se_2_] (^57^2)

This compound was prepared using a modification of the preparation of non-isotopically labelled **2** given above. A solution of SePMe_3_ (0.0318 g, 0.205 mmol) in THF (3 ml) was added to a solution of L_2_^57^Fe_2_(N_2_) (0.0987 g, 0.101 mmol) in THF (3 ml). After addition, the mixture was stirred overnight at room temperature. The resulting dark-brown mixture was filtered through a glass frit and the resulting dark-green solid washed with additional THF (4 ml). The dark-green solid was then dried under vacuum to afford 0.0441 g (39%) of the isotopically labelled crude diferric complex ^**57**^**2**^**ox**^ (L_2_^57^Fe_2_Se_2_). Solid KC_8_ (0.0068 g, 0.050 mmol) was added to a stirred suspension of the isotopically labelled crude diferric complex (0.0441 g, 0.040 mmol) in THF (3 ml). The mixture was stirred for 1 h, at which point the dark-green precipitate had been consumed to afford a dark-brown mixture. The mixture was filtered through a glass microfibre filter pad to remove graphite, rinsing with additional THF (~3 ml). The dark-brown filtrate was then concentrated under vacuum. The residue was triturated with Et_2_O (3 ml) and filtered through a glass microfibre filter pad. The resulting black solid was washed with additional Et_2_O until the filtrate ran clear (~4 ml). The black solid was then resolubilized in THF (~1 ml), filtered through the glass microfibre pad, further concentrated to half-volume under vacuum and stored at –35 °C to afford 0.0081 g (12% yield for reduction step, 5% overall yield) of black crystals of the isotopically labelled ^**57**^**2**.

### Infrared and UV-vis

Infrared spectra (400–4,000 cm^−1^) of solid samples were recorded on a Thermo Scientific Nicolet iS5 FT-IR spectrometer equipped with an iD7 attenuated total reflectance device using a diamond cell. Solution UV-vis spectra (250–1,100 nm) were recorded on an Agilent Technologies Cary 8454 UV-vis spectrometer in quartz cuvettes with a 1-cm pathlength.

### X-ray crystallography

The crystal structures of compounds **2**^**ox**^, **2**, **3**^**ox**^, **3**′ and **3**″ were determined using either a Bruker AXS Enraf-Nonius or Bruker D8 Venture Kappa diffractometer equipped with a Mo IμS anode and INCOATEC Helios mirror optics (*λ* = 0.71073 Å). Diffraction data were collected at 100 K (200 K for **2**) in a nitrogen cryostream. Final cell constants were obtained from least-squares fits of several thousand strong reflections. The intensities of redundant reflections were used to correct for absorption using the SADABS program^52^. The structures were readily solved by Patterson methods and subsequent difference Fourier techniques. The Siemens ShelXTL software package^53^ was used for solution of the structures, and ShelXL-2013 (ref. ^54^) was used for structure refinement. All non-hydrogen atoms were anisotropically refined, and hydrogen atoms bound to carbon were placed at calculated positions and refined as riding atoms with isotropic displacement parameters. The crystal structures presented in the manuscript and Supporting Information were generated using the Olex2 software^55^. CCDC 1920937, 2077197, 2077198, 2077199 and 2077200 contain the supplementary crystallographic data for this paper. These data can be obtained free of charge from The Cambridge Crystallographic Data Centre via www.ccdc.cam.ac.uk/data_request/cif. The crystallographic refinement details for compounds **2**^**ox**^, **2**, **3**^**ox**^, **3**′ and **3**″ are collected in Supplementary Table 2.

### Powder X-ray diffraction

The powder X-ray diffraction patterns for qualitative phase analysis were collected on a Stoe STADI P transmission diffractometer using Mo radiation (0.7093 Å). The instrument was equipped with a primary Ge(111) monochromator (Mo Kα_1_) and a position-sensitive Mythen1K detector. Data were collected in the 2*θ* range 2–30° with a step width of 0.015°. The measuring time per step was 10 s. One, two or four scans were collected for each sample and summed after data collection. Low-temperature data (200 K) were obtained by cooling using an Oxford Cryostream 700 instrument. Samples were added to glass capillaries (diameter 0.5 mm) in the glove box and sealed with silicone grease and a rubber septum. For measurement at 200 K, the capillary was flame-sealed. The measured patterns were evaluated qualitatively by comparison with crystal structure data from single-crystal refinement.

### SQUID magnetometry

Temperature-dependent (2–290 K) and field-dependent (2 K) magnetic susceptibility data were recorded on a SQUID magnetometer (MPMS Quantum design) in external magnetic fields ranging from 0.1 to 7 T. The experimental susceptibility data were corrected for underlying diamagnetism by using tabulated Pascal’s constants. Standard deviations for experimental values are within the radius of the data points, represented as open circles in Fig. 3b,c and the Supplementary Information, unless indicated otherwise.

### Mössbauer spectroscopy

^57^Fe Mössbauer spectra were recorded on conventional spectrometers with alternating constant acceleration of the γ source. The minimum experimental line width was 0.24 mm s^–1^ (fwhm). The sample temperature was maintained constant in either an Oxford Instruments Variox cryostat or a cryogen-free, closed-cycle Mössbauer magnet cryostat from Cryogenic. The latter consists of a split-pair superconducting magnet system for applied fields up to 7 T, with the field at the sample perpendicular to the γ beam. The ^57^Co/Rh source (1.8 GBq) was positioned at room temperature inside the gap of the magnet system at the zero-field position by using a re-entrant bore tube. The detector was an Ar/10% CH_4_-filled end-window-type proportional counter for the zero-field measurements and a Si drift diode (150 mm^2^ SDD CUBE) of an AXAS-M1 system from Ketek with a vacuum-tight 200 mm stainless-steel finger, which was inserted into the cryostat to position the diode also in the gap of the magnet. Isomer shifts are quoted relative to Fe metal at 300 K. Zero-field Mössbauer spectra were fitted with Lorentzian doublets using the program *mf* (version 2.2 (universal); July 2014; E. Bill, Max Planck Institute for Chemical Energy Conversion, D-45470 Mülheim an der Ruhr). All parameters are reported in Supplementary Table 3. The magnetic Mössbauer spectra were simulated using the program *mx* (version 2.0; February 2011; E. Bill, Max Planck Institute for Chemical Energy Conversion, D-45470 Mülheim an der Ruhr). All parameters are reported in Supplementary Table 5.

### EPR spectroscopy

The continuous-wave X-band (~9.63 GHz) EPR spectra of **1**–**3** frozen in acetone were measured on a Bruker E500 spectrometer equipped with an Oxford liquid helium flow cryostat. The spectra were collected in a dual-mode X-band resonator, operated in perpendicular mode (TE_102_). All spectra were collected with 100 kHz field modulation at 6 G amplitude. All continuous-wave EPR spectra were simulated in Matlab 2020b with the EasySpin (v 6.0.0-dev29) package^56^. A Bruker Elexsys-580 spectrometer equipped with a split-ring resonator and an Oxford C-935 liquid helium cryostat was used to perform spin nutation experiments on frozen solution samples of **1** and **2** in 2-methyltetrahydrofuran at 5.5 K with the following pulse sequence: *t*_p_–*T*–π/2–*τ*/2–π–*τ*–echo, *T* = 1,000 ns, *τ* = 300 ns, π/2 = 16 ns, *t*_p_ = 2 ns increased in 2 ns step size, 100 µs repetition rate and 1,024 shots per point. After a nutation pulse, *t*_p_, the longitudinal magnetization was indirectly detected at a time *T* > *t*_p_ to ensure complete decay of the electron coherence. Varying the repetition rate revealed the phase memory, *T*_M_, to be considerably faster than the lower spectrometer limit of 100 µs. Two-pulse electron spin echo envelope modulation experiments exhibited full echo decay by ~300 ns (upper limit of *T*_M_). Nutation frequencies, *Ω*_nut_, follow the equation ${{\Omega}}_{{\mathrm{nut}}} = \frac{{g_1\beta _{\mathrm{e}}B_1}}{\hbar }\left[ {S\left( {S + 1} \right)} \right]^{\frac{1}{2}}$, where *g*_1_ is the *g* value in the laboratory frame, *β*_e_ is the Bohr magneton, *B*_1_ is the microwave power and ℏ is the reduced Planck’s constant. All Fourier transforms were obtained by standard treatment of the time-domain spectra, including subtraction of a cubic baseline, Hann windowing, zero filling and fast-Fourier transform. Q-band (~35 GHz) ^57^Fe pulsed ENDOR spectra were collected on a custom-built instrument equipped with a liquid helium immersion Dewar at 2 K at Northwestern University (Evanston, IL, USA) ^57,58^. Data acquisition was performed with the SpecMan software package^59^ (http://specman4epr.com) in conjunction with a Spin-Core PulseBlaster ESR PRO 400 MHz word generator and Agilent Technologies Acquiris DP235 500 MS s^−1^ digitizer. Collection of the two-dimensional field–frequency ^57^Fe ENDOR pattern was achieved through the use of the Davies ENDOR sequence, π–*T*_RF_–π/2–*τ*–π–*τ*–echo, where RF is the applied time *T*_RF_. Each ENDOR spectrum was acquired at a static magnetic field for a minimum of 100 scans at a repetition rate of 50 ms with pulse parameters of π/2 = 80 ns, *τ* = 600 ns and *T*_RF_ = 35 µs.

### X-ray emission spectroscopy

The Fe Kβ X-ray emission spectra of complexes **1** and **2** were measured at separate synchrotron beamlines (SOLEIL GALAXIES for **1** and the European Synchrotron Radiation Facility (ESRF) ID-26 for **2**), whereas complex **3** was measured on the in-house LabXES spectrometer^60^, which has approximately twofold lower resolving power than the synchrotron spectrometers (*E*/Δ*E* ≈ 3,900 at 7.06 keV for LabXES^60^ compared with 8,800 at ID26; ref. ^61^). The X-ray emission spectrum for complex **1** has been previously reported^27^. Sample preparation and experimental details for complexes **2** and **3** are described below. The Fe Kβ X-ray emission spectrum of complex **2** was measured at the ID26 beamline (6 GeV, 90 mA, 16-bunch mode) of the ESRF equipped with a liquid helium cryostat and sample changer operated at 20 K. A Si(111) double-crystal monochromator was used upstream for energy selection and calibrated to the first inflection point of an Fe foil set to 7,111.2 eV. The beam size was 600  × 70 μm, providing a flux density of ~1 × 10^12^ photons s^–1^. A 1-m-radius Johann-type X-ray emission spectrometer was used, equipped with five spherically bent Ge(620) analyser crystals. The nominal analyser Bragg angle for Fe Kβ_1,3_ was 73.1°. The X-ray emission spectrometer was internally calibrated using the emission lines of Fe_2_O_3_ (Kβ_1,3_: 7,059.4 eV; Kβ′: 7,044.9 eV). Data were acquired using a dead-time-corrected silicon drift diode detector (Ketek) aligned on the Rowland circle. Possible attenuation of the fluorescence signal was reduced by placing a helium-filled flight path between the sample, the analyser crystals and detector. Incident excitation energy was selected well above the absorption edge at 7.8 keV. Sample **2** was diluted in boron nitride to approximately 2% Fe by mass and prepared in an 1-mm aluminium spacer with 38-μm-thick Kapton tape windows. The Fe Kβ X-ray emission spectrum of complex **3** was measured on LabXES. To avoid spectral broadening due to incident-beam penetration effects, **3** was prepared as a thin powder spread on the 38-μm-thick Kapton tape window of a polyether ether ketone cell. Aluminium filters (800 nm) were used to attenuate UV-vis fluorescence. To minimize radiation damage, the sample was mounted on a helium displex cryostat with a base temperature of 11–14 K, although the temperature at the sample was estimated to be 60–80 K. LabXES uses an Excillum gallium metal jet X-ray tube to obtain a high-incident photon flux and a full-cylinder von Hamos geometry to maximize the solid angle of detection. The source was operated at 250 W, the detector was in the post-focus position and the total collection time was 18 h. The sample and spectrometer chamber were kept at pressures of 10^–7^–10^–6^ mbar to minimize signal attenuation. A single-photon counting algorithm with manually chosen energy windows was used to reject signals from other elements, and a linear subtraction was applied to remove the background due to randomly oriented photons reflected by the spectrometer chamber.

### DFT calculations

All geometry optimizations, single-point and frequency calculations were executed using ORCA^62,63^ (version 4.1). Computations were performed using the hybrid TPSSh^64,65^ functional with the atom-pairwise dispersion correction with the Becke-Johnson damping scheme (D3BJ)^66,67^ dispersion correction and conductor-like polarizable continuum model (CPCM)^68–70^ solvation model. The zeroth-order regular approximation (ZORA)^71,72^ relativistic approximation was used and employed the relativistically contracted def2 Ahlrichs^73,74^ basis set. A triple-ζ ZORA-def2-TZVP basis set was used for all Se, Fe, S and N atoms, with ‘old-ZORA-TZVP’ used for Te, and a double-ζ def2-SVP basis set used for all other atoms. The resolution of identity approximation for Coulomb integrals and numerical chain-of-spheres integration for the Hartree-Fock Exchange integrals (RIJCOSX)^75,76^ were used to speed up the calculations. For the complexes discussed in this work, appropriate antiferromagnetic ground states were achieved starting from a ‘high-spin’ ferromagnetic solution and employing spin-flip to access the broken-symmetry solution. Crystal structures were used as the starting point for all geometry optimization calculations. Heisenberg exchange coupling values were calculated using the Yamaguchi spin projection method^77,78^. Mössbauer isomer shifts were calculated using previously outlined protocols^79^. PKS vibrational frequencies were determined through numerical frequency calculations on the broken-symmetry solutions (*S* = 1/2) and visualized using Jmol^80^.

## Online content

Any methods, additional references, Nature Research reporting summaries, source data, extended data, supplementary information, acknowledgements, peer review information; details of author contributions and competing interests; and statements of data and code availability are available at 10.1038/s41557-021-00853-5.

## Supplementary information

Supplementary Information Supplementary Discussion, Figs. 1–37 and Tables 1–11.

Supplementary Data 1 Crystal structure of complex **2**, CCDC 1920937.

Supplementary Data 2 Crystal structure of complex **2**^**ox**^, CCDC 2077197.

Supplementary Data 3 Crystal structure of complex **3**′, CCDC 2077199.

Supplementary Data 4 Crystal structure of complex **3**″, CCDC 2077200.

Supplementary Data 5 Crystal structure of complex **3**^**ox**^, CCDC 2077198.

## Data Availability

Crystallographic data for the structures in this article and Supplementary Information have been deposited at the Cambridge Crystallographic Data Centre under deposition numbers 2077197 (**2**^**ox**^), 1920937 (**2**), 2077198 (**3**^**ox**^), 2077199 (**3**′) and 2077200 (**3**″). Copies of data can be obtained free of charge from https://www.ccdc.cam.ac.uk/structures/. Other data that support the findings of this study can be found in the article and Supplementary Information. All spectroscopic data, minimal processing scripts and optimized geometry coordinates are available freely online^81^.
