# Supplementary material for: Stabilization of intermediate spin states in mixed-valent diiron dichalcogenide complexes
Source: Nat Chem. 2022 Jan 20;14(3):328–33. doi: 10.1038/s41557-021-00853-5 (PMC8898764; doi:10.1038/s41557-021-00853-5)
Supplement: Supplementary file 1 — Supplementary Discussion, Figs. 1–37 and Tables 1–11. [file 41557_2021_853_MOESM1_ESM.pdf]

nature portfolio

https://doi.org/10.1038/s41557-021-00853-5

Supplementary information

Stabilization of intermediate spin states in mixed-valent diiron dichalcogenide complexes

In the format provided by the authors and unedited

Stabilization of Intermediate Spin-States in Mixed-valent Diiron Dichalcogenide Complexes

Justin T. Henthorn[1*], George E. Cutsail III[1,2*], Thomas Weyhermüller[1], and Serena DeBeer[1]

Supplementary Information

[1]Max Planck Institute for Chemical Energy Conversion, Stiftstr. 34-36, Mülheim an der Ruhr D-45470, Germany.

[2]Institute for Inorganic Chemistry, University of Duisburg- Essen, Universitätsstr. 5-7, D-45117 Essen, Germany

[*] Correspondence to: justin.henthorn@cec.mpg.de, george.cutsail@cec.mpg.de

Table of Contents

Supplemental Figures 1-4	3
Supplemental Tables 1-2	5
Supplemental Figures 5-11	7
Supplemental Table 3	13
Supplemental Figures 12-14	13
Variable Temperature Zero-field Mössbauer Discussion	15
Supplemental Figure 15	16
Inter-valence Charge Transfer Discussion	16
Supplemental Figures 16-17	17
Discussion of Limitations of the HDE Model to Magnetic Data Fitting	19
Supplemental Table 4	20
Supplemental Figures 18-31	21
Supplemental Table 5	33
Supplemental Figures 32-35	34
Supplemental Tables 6-10	38
Supplemental Figure 36	40
Incorporation of Vibronic Coupling into the Spin Ladder Energy	41
Supplemental Table 11	41
Supplemental Figure 37	42
Supplemental References	43

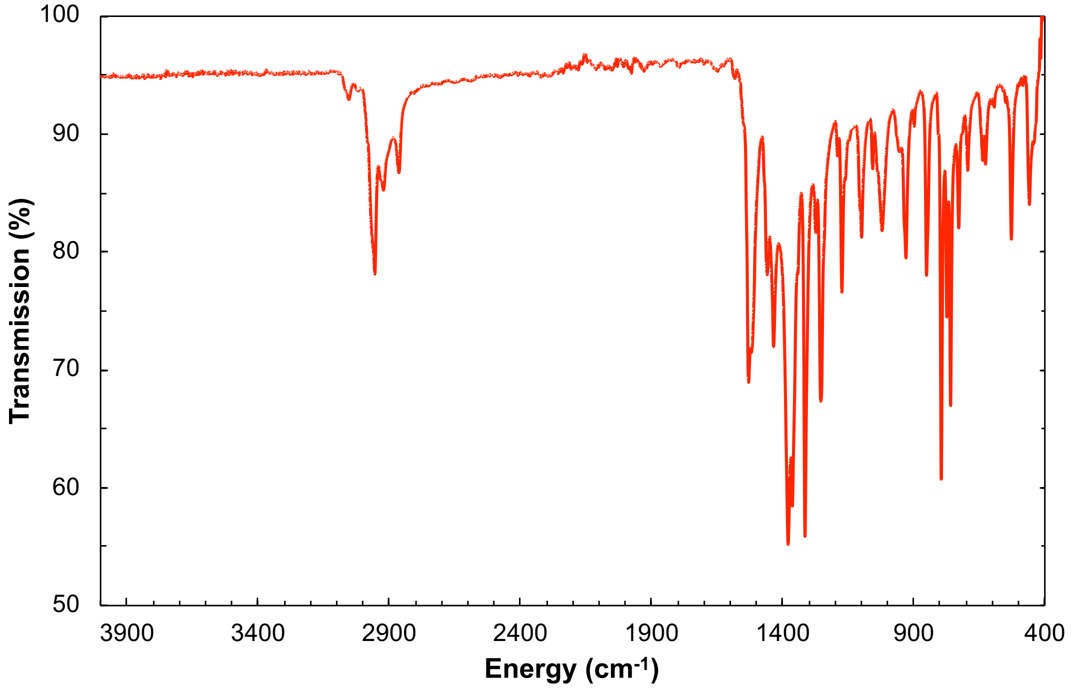

Figure 1. ATR-IR spectrum of $\mathbf{2}^{ox}$.

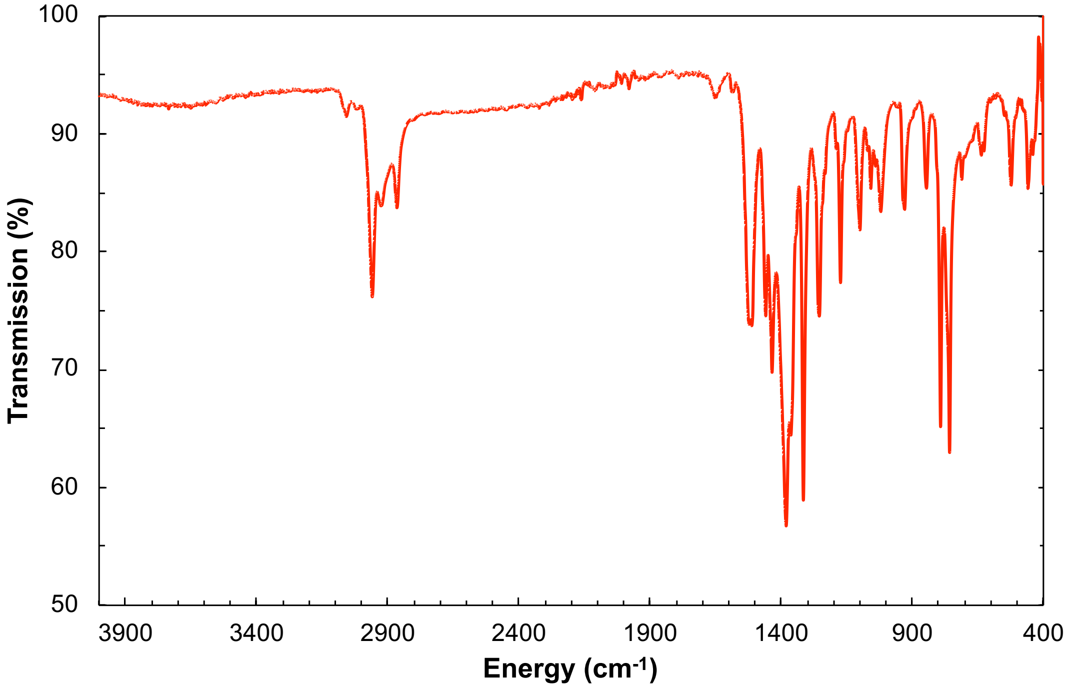

Figure 2. ATR-IR spectrum of **2**.

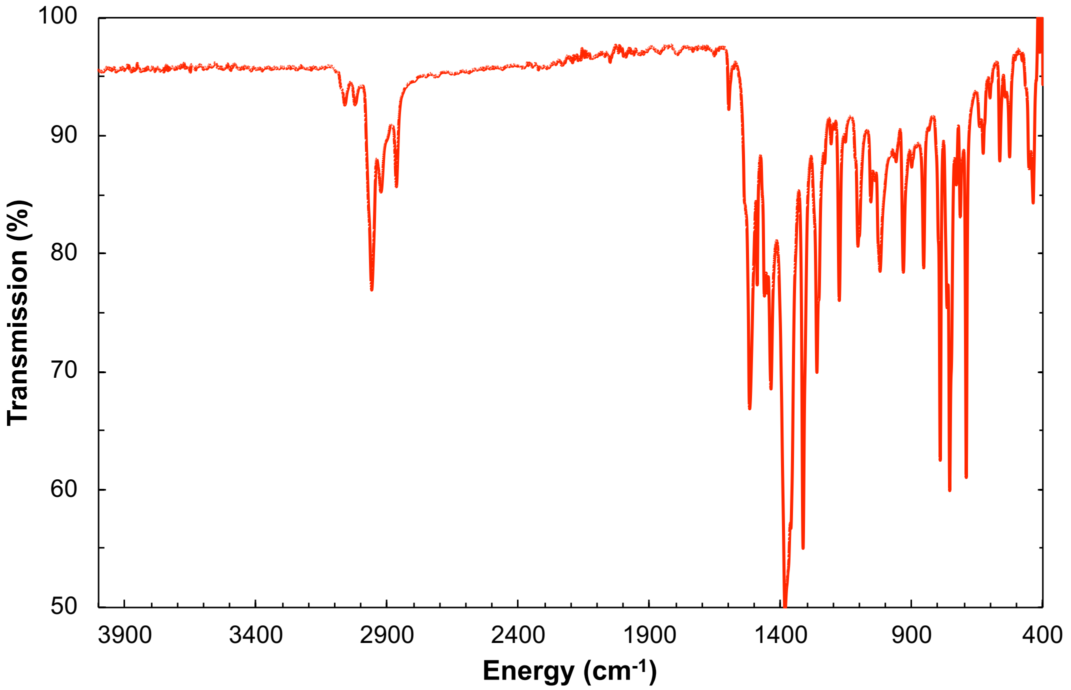

Figure 3. ATR-IR spectrum of solid **3**^{ox}.

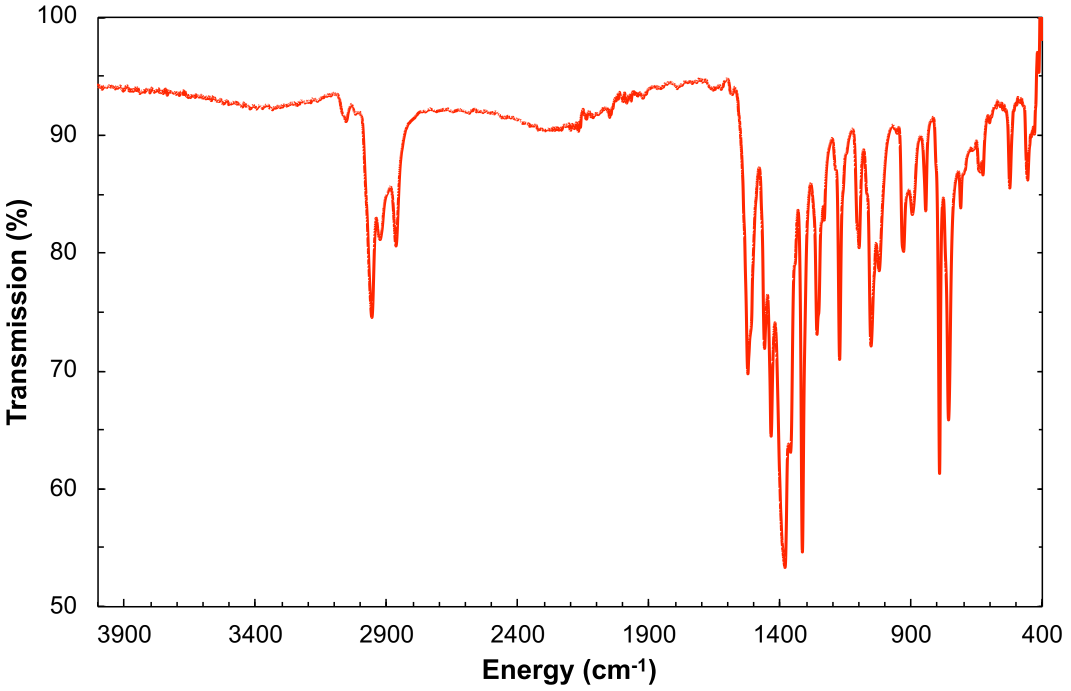

Figure 4. ATR-IR spectrum of solid **3**.

Table 1. Selected bond lengths and angles in complexes **1**^{ox}-**3**^{ox} and **1-3''**.

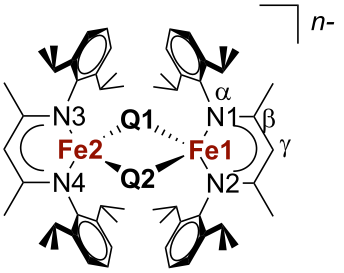

	**1ox**	**1**	**2ox**	**2**[a]
Fe1–Fe2 (Å)	2.8159(7)	2.8071(6)	2.9427(6)	2.9660(8)
Fe1–Q1 (Å)	2.2130(8)	2.2379(8)	2.3351(7)	2.3607(7)
Fe1–Q2 (Å)	2.2056(7)	2.2394(7)	2.3307(5)	2.3659(9)
Fe2–Q1 (Å)	2.2056(7)	2.2394(7)	2.3307(5)	2.3659(9)
Fe2–Q2 (Å)	2.2130(8)	2.2379(8)	2.3351(7)	2.3607(7)
∠Fe1-Q1-Fe2 (°)	79.18(2)	77.66(2)	78.20(2)	77.73(2)
∠Fe1-Q2-Fe2 (°)	79.18(2)	77.66(2)	78.20(2)	77.73(2)
Fe1–N1 (Å)	2.027(2)	2.101(2)	2.036(2)	2.067(3)
Fe1–N2 (Å)	2.029(2)	2.096(2)	2.033(2)	2.075(3)
Fe2–N3 (Å)	2.029(2)	2.096(2)	2.033(2)	2.075(3)
Fe2–N4 (Å)	2.027(2)	2.101(2)	2.036(2)	2.067(3)

	**3ox**[b]		**3'**[b]		**3''**[c]
Fe1–Fe2 (Å)	3.0562(5)	3.0902(4)	3.1668(6)	3.1521(6)	3.1978(5)
Fe1–Q1 (Å)	2.5312(3)	2.5341(3)	2.5687(6)	75.50(2)	2.5792(7)
Fe1–Q2 (Å)	2.5250(3)	2.5298(3)	2.5603(5)	2.6010(6)	2.6325(7)
Fe2–Q1 (Å)	2.5250(3)	2.5298(3)	2.5603(5)	2.6010(6)	2.5645(7)
Fe2–Q2 (Å)	2.5312(3)	2.5341(3)	2.5687(6)	2.5472(6)	2.5438(7)
∠Fe1-Q1-Fe2 (°)	74.38(1)	75.214(9)	76.26(2)	75.50(2)	76.88(2)
∠Fe1-Q2-Fe2 (°)	74.38(1)	75.214(9)	76.26(2)	75.50(2)	76.29(2)
Fe1–N1 (Å)	2.0239(7)	2.0309(7)	2.062(3)	2.069(6)	2.076(2)
Fe1–N2 (Å)	2.0239(7)	2.0309(7)	2.061(3)	2.051(4)	2.072(2)
Fe2–N3 (Å)	2.0239(7)	2.0309(7)	2.061(3)	2.051(4)	2.063(2)
Fe2–N4 (Å)	2.0239(7)	2.0309(7)	2.062(3)	2.069(6)	2.049(2)

[a]Diffraction data for **2** was collected at 200K, all other structures were collected at 100K (**Supplemental Table 2**). [b]The unit cells of **3**^{ox} and **3'** each exhibit two crystallographically distinct $[L_2Fe_2Te_2]^{n-}$ moieties. [b]The potassium ion is bound to Te2 (Q2) as well as the γ-carbon and the N1-bound β-carbon of the N1/N2 β-diketiminate ligand.

Table 2. Crystallographic refinement details for complexes **2**^{ox}, **3**^{ox}, **2**, **3′** and **3″**.

Compound	**2**^{ox}	**3**^{ox}•1.5PhMe	**2**	**3′**	**3″**
CCDC	2077197	2077198	1920937	2077199	2077200
empirical formula	$C_{58}H_{82}Fe_2N_4Se_2$	$C_{68.5}H_{94}Fe_2N_4Te_2$	$C_{90}H_{146}Fe_2KN_4O_8Se_2$	$C_{90}H_{146}Fe_2KN_4O_8Te_2$	$C_{66}H_{98}Fe_2KN_4O_2Te_2$
formula wt	1104.89	1340.37	1720.82	1818.10	1385.52
T (K)	100	100	200	100	100
a, Å	22.464(2)	18.570(2)	19.040(4)	13.125(1)	24.754(2)
b, Å	14.807(2)	20.977(2)	18.815(4)	17.983(2)	14.029(1)
c, Å	16.386(3)	16.449(2)	26.235(5)	19.525(2)	22.214(2)
alpha, deg	90	90	90	91.581(4)	90
beta, deg	90.07(1)	99.235(2)	92.30(3)	90.544(4)	122.528(2)
gamma, deg	90	90	90	92.274(4)	90
V, $Å^3$	5450.4(1)	6325(1)	9391(3)	4602.9(8)	6504.2(9)
Z	4	4	4	2	4
crystal system	Monoclinic	Monoclinic	Monoclinic	Triclinic	Monoclinic
space group	C2/c	C2/m	C2/c	P-1	Cc
d_{calc}, g/cm^3	1.346	1.408	1.217	1.312	1.415
θ range, deg	2.982-33.169	2.312-37.013	2.595-28.500	1.862-30.000	1.76-35.00
μ, mm^{-1}	1.908	1.406	1.181	1.035	1.434
abs cor	Gaussian	Gaussian	Gaussian	Multi-scan	Numerical
GOF	1.046	0.730	1.067	1.086	1.062
R_1,^{a} wR_2^{b} (I > 2sig(I))	0.0445, 0.1199	0.0186, 0.0849	0.0613, 0.1531	0.0584, 0.1443	0.0237, 0.0621

^{a} $R_1 = \sum\big||F_o| - |F_c|\big| / \sum|F_o|$. ^{b} $wR_2 = [\sum[w(F_o^2 - F_c^2)^2] / \sum[w(F_o^2)^2]]^{1/2}$

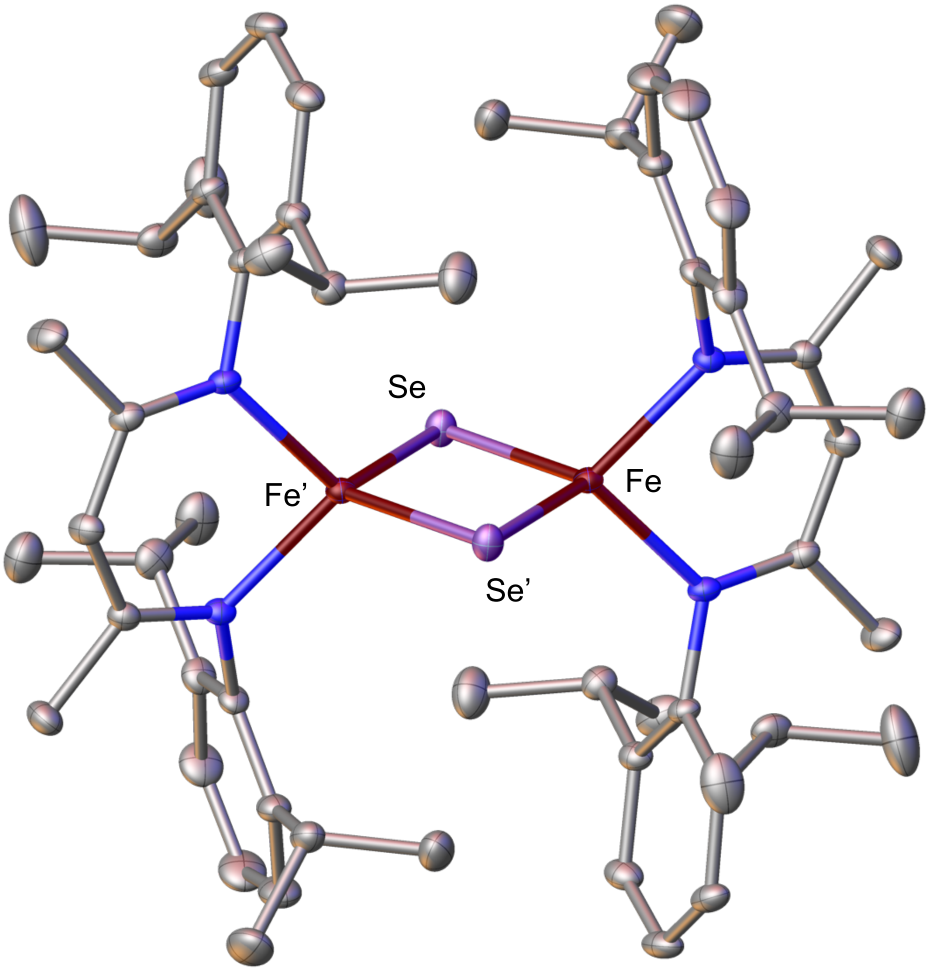

Figure 5. Structural drawing of **2**^{ox} (CCDC 2077197) with 50% probability ellipsoids. Carbon atoms are shown in grey, nitrogen atoms in blue. Hydrogen atoms omitted for clarity.

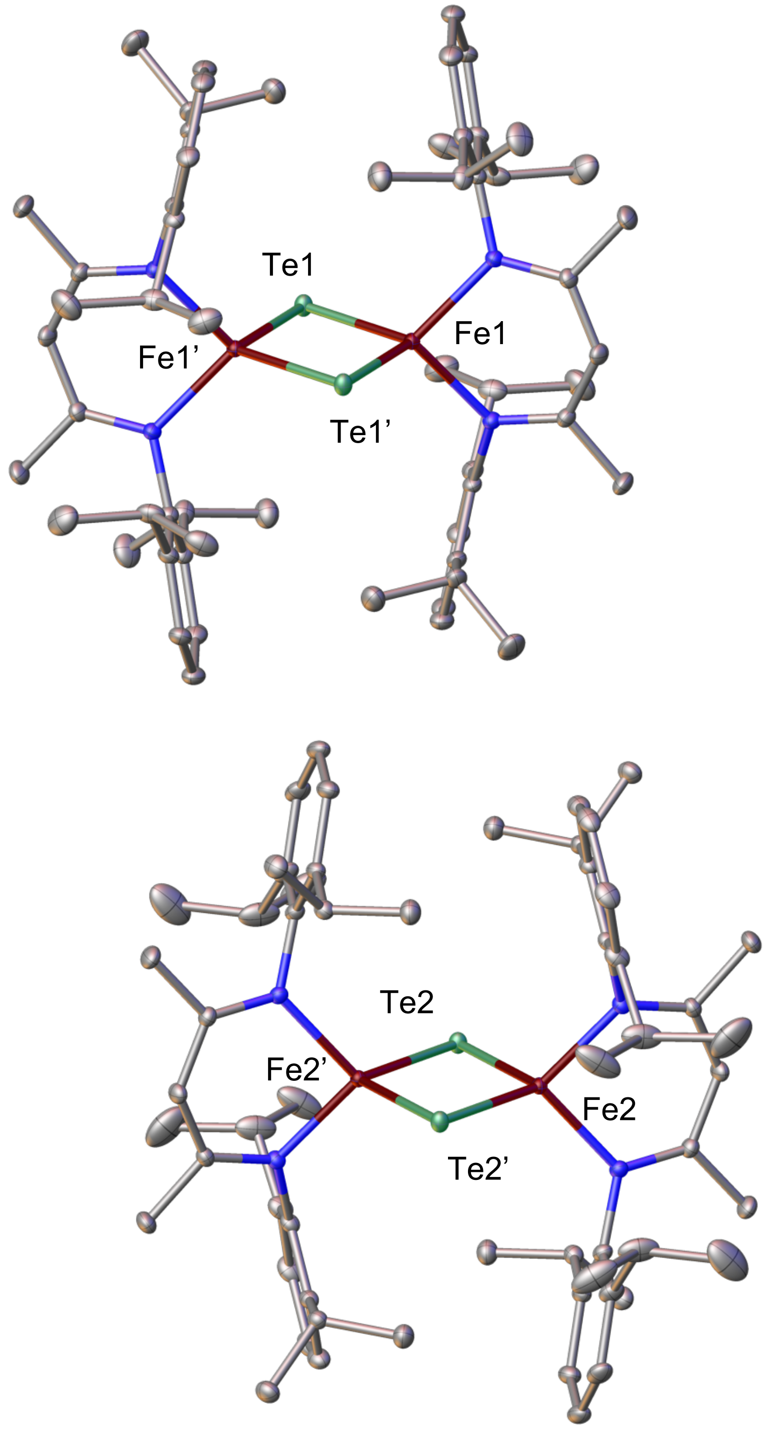

Figure 6. Structural drawing of **3^{ox}•1.5PhMe** (CCDC 2077198) with 50% probability ellipsoids. Carbon atoms are shown in grey, nitrogen atoms in blue. Hydrogen atoms and solvent molecules are omitted for clarity. Both crystallographically distinct $L_2Fe_2Te_2$ units are shown.

Special refinement details for 3^{ox}•1.5PhMe: The PhMe solvent molecules were positionally disordered and satisfactorily modelled using PART, EADP, and ISOR cards in ShelX.

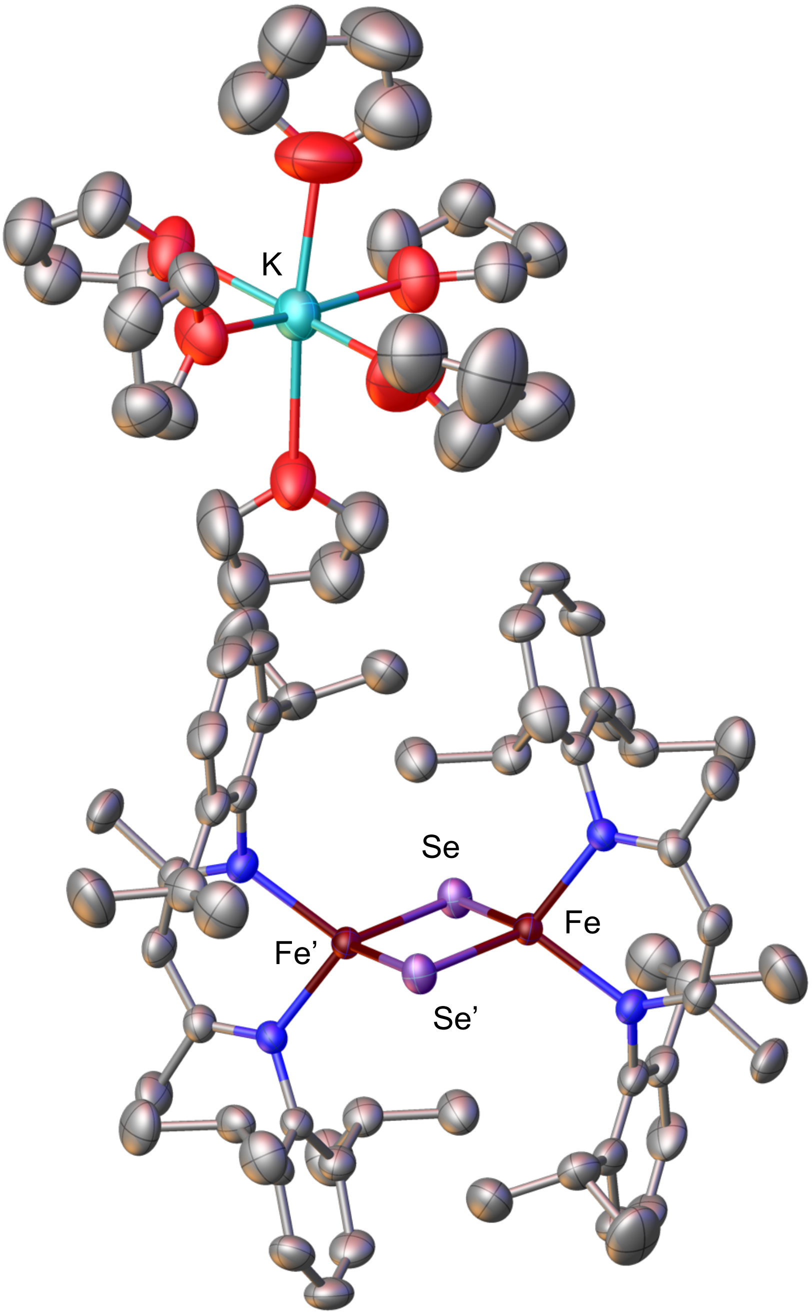

Figure 7. Structural drawing of **[K(THF)$_6$][L$_2$Fe$_2$Se$_2$]•2THF** (**2**, CCDC 1920937) with 50% probability ellipsoids. Carbon atoms shown in grey, nitrogen in blue, and oxygen in red. Hydrogen atoms and outer-sphere THF molecules omitted for clarity.

Special refinement details for 2: All four crystallographically distinct THF solvent molecules were positionally disordered and satisfactorily modelled using PART, EADP, and ISOR cards in ShelX.

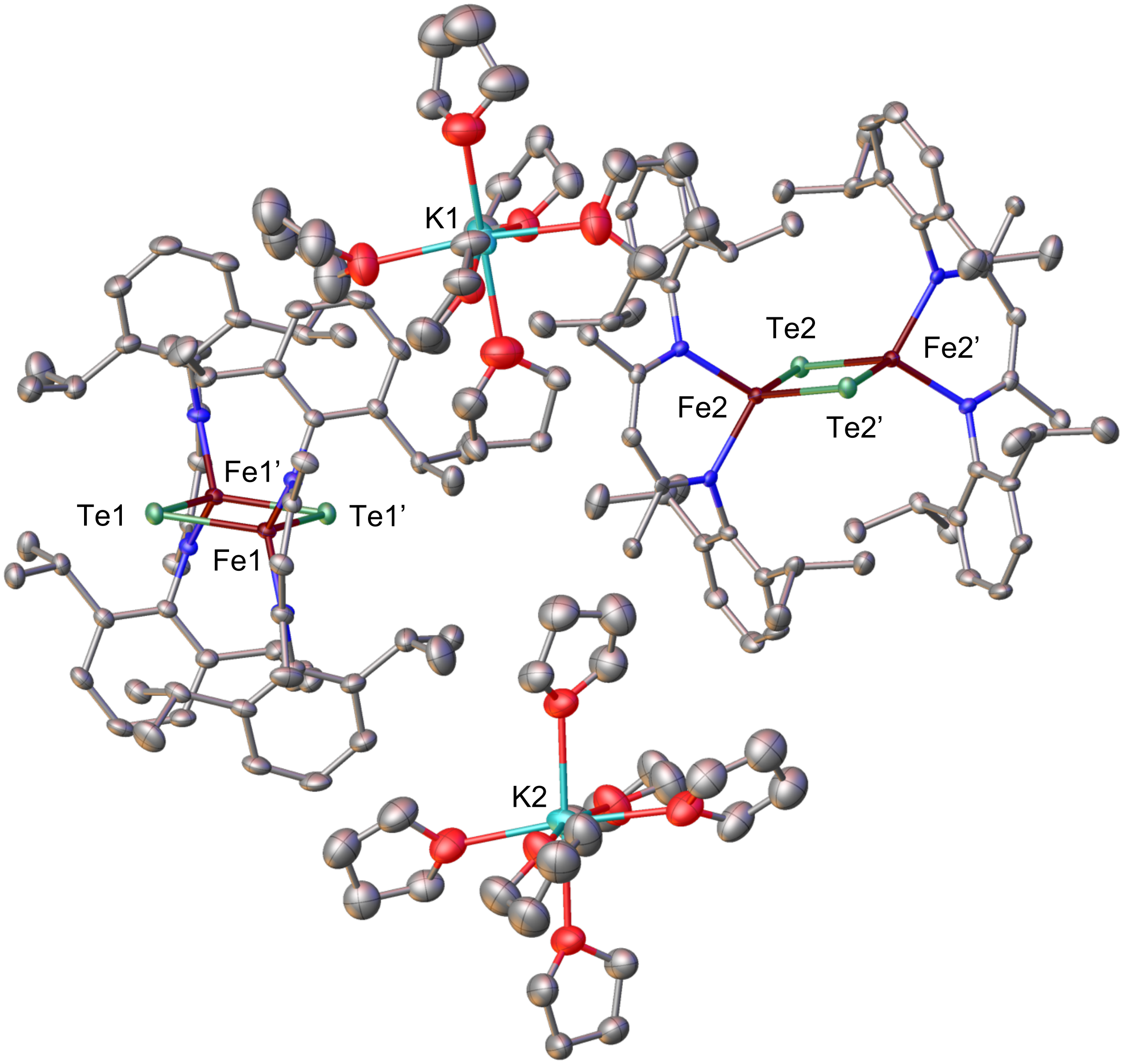

Figure 8. Structural drawing of **[K(THF)$_6$][L$_2$Fe$_2$Te$_2$]•2THF** (**3'**, CCDC 2077199) with 50% probability ellipsoids. Carbon atoms are shown in grey, nitrogen atoms in blue, and oxygen atoms in red. Hydrogen atoms and outer-sphere THF molecules omitted for clarity. Both crystallographically distinct [L$_2$Fe$_2$Te$_2$][K(THF)$_6$] pairs are shown.

Special refinement details for 3': One of the two [L$_2$Fe$_2$Te$_2$]$^-$ fragments in the asymmetric unit and the free THF solvent molecules were positionally disordered and satisfactorily modelled using PART, EADP, ISOR, SIMU, SADI, and EQIV cards in ShelX.

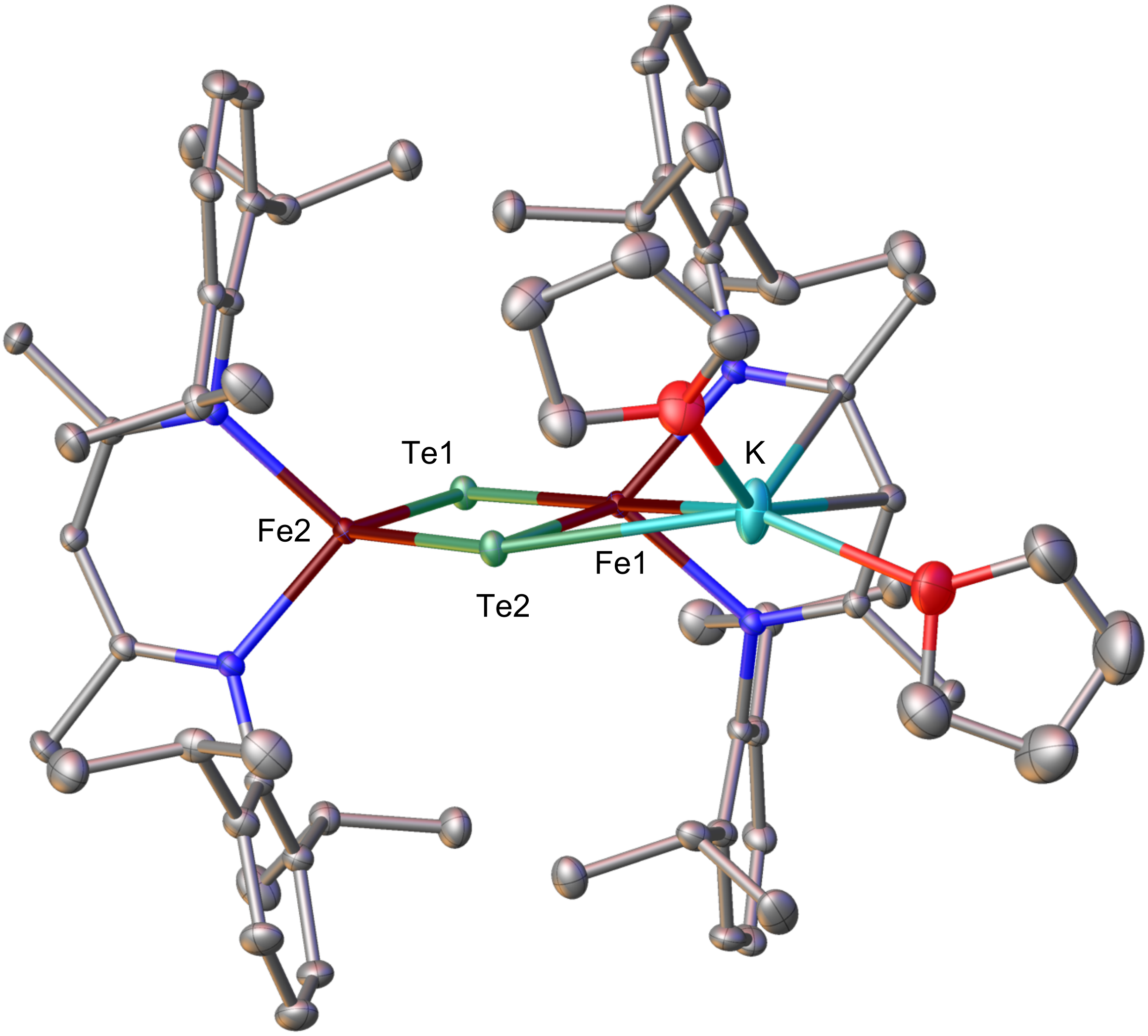

Figure 9. Structural drawing of $\mathbf{L_2Fe_2Te_2K(THF)_2}$ (**3''**, CCDC 2077200) with 50% probability ellipsoids. Carbon atoms are shown in grey, nitrogen atoms in blue, and oxygen atoms in red. Hydrogen atoms omitted for clarity.

Special refinement details for 3'': Refined as a two-component inversion twin with twin law (-1 0 0 0 -1 0 0 0 -1) and BASF 0.403(9).

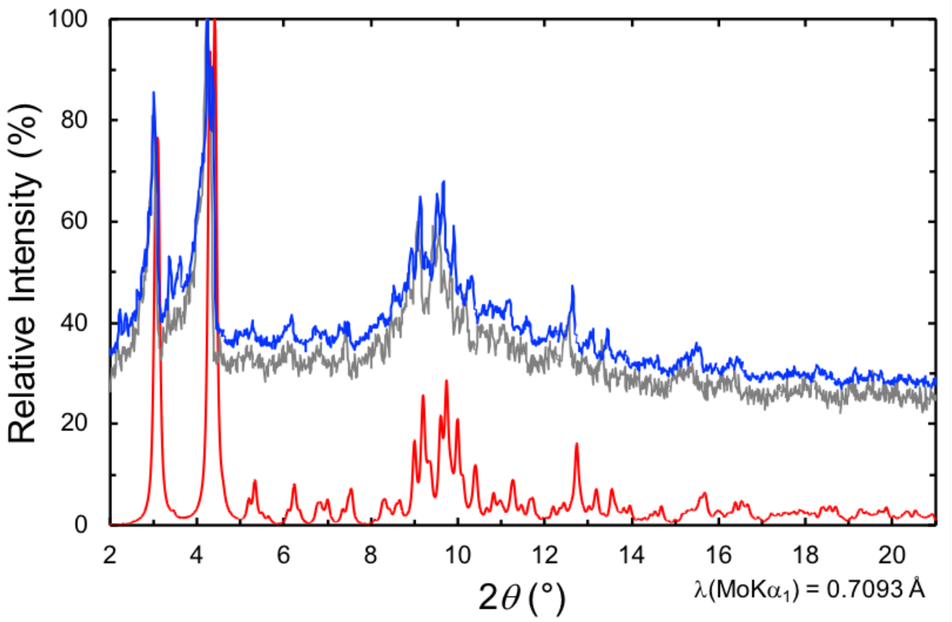

Figure 10. Comparison of powder XRD of **2-[Fe_2Se_2]$^+$** at 200 K (blue) and 298 K (grey) to calculated XRD based on single crystal data at 200 K (red). The two small sets of sharp features at ca 2.5 and 3.5° in the 200 K powder spectrum (blue) are assigned to decomposition during flame-sealing of the capillary, as preliminary room temperature measurements (grey) prior to flame sealing do not exhibit these features.

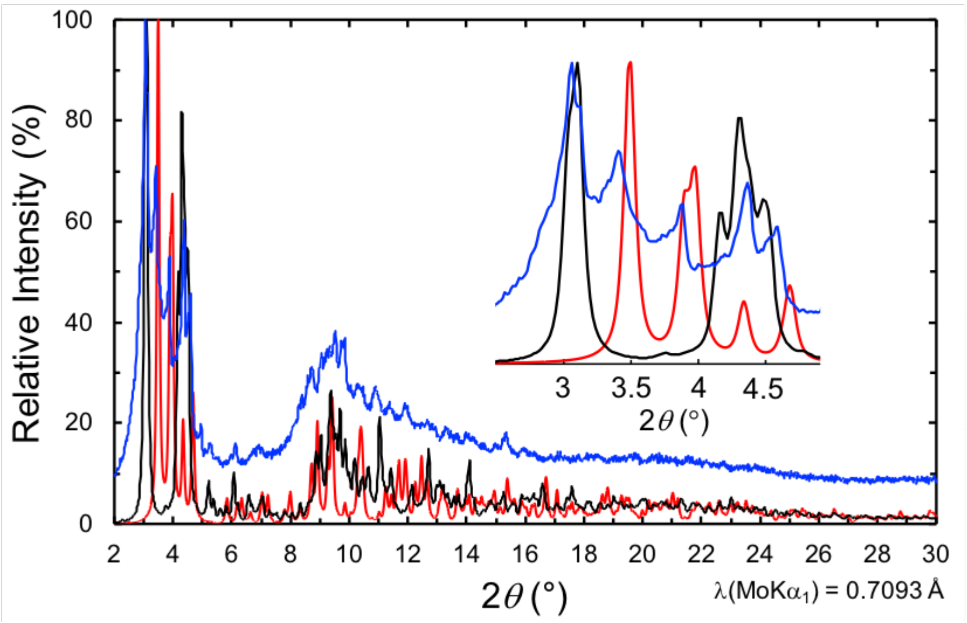

Figure 11. Comparison of powder XRD of **3-[Fe_2Te_2]$^+$** (blue) to calculated XRD based on single crystal data of **3'** (red) and **3''** (black). Powder XRD was collected at 298 K while single crystal data were both collected at 100 K. Inset: low-angle data comparison. The data is consistent with a mixture of structures **3'** and **3''**.

Table 3. Zero-field ^{57}Fe Mössbauer (80 K) fitting parameters for complexes **1**^{ox} – **3**^{ox} and **1** – **3**.

Compound	δ[a] (mm/s)	$\lvert\Delta E_Q\rvert$ (mm/s)	Fwhm[b] (mm/s)	η[c] (a.u.)	relative intensity (%)
**1**^{ox}	0.34[d]	1.15[d]	0.28[d]	1[d]	92[d]
	0.99[d,e]	1.87[d,e]	0.41[d,e]	1[d,e]	8[d,e]
**2**^{ox}	0.38	0.99	0.26	1	100
**3**^{ox}	0.43	0.70	0.46	1	100
**1**	0.40[d]	1.01[d]	0.34[d]	1[d]	51[d]
	0.76[d]	1.21[d]	0.39[d]	1.27[d]	49[d]
**1**[f]	0.53[f]	0.74[e]	0.39[f]	0.73[f]	44[f]
	0.63[f]	1.45[f]	0.39[f]	1.42[f]	56[f]
**2**	0.44	0.88	0.39	1	54
	0.78	1.35	0.28	1.55	46
**3**	0.68	1.23	0.45	1.04	100

[a]Estimated standard deviation ± 0.02 for isomer shift, quadrupole splitting, and Fwhm, and ± 0.05 for relative intensity. [b]Full width at half-maximum. [c]Asymmetry parameter. [d]Data originally reported in reference [1]. [e]Unidentified ferrous impurity. [f]Alternate nested fitting for **1**.

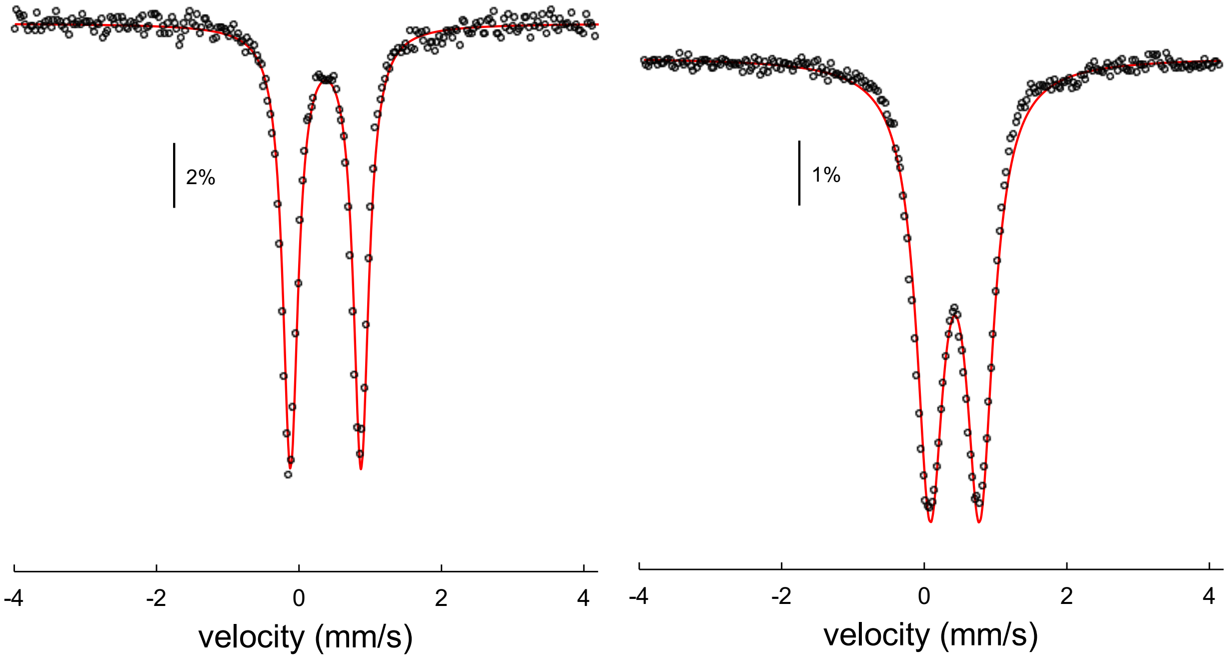

Figure 12. Mössbauer spectrum of solid **2**^{ox} (left) and **3**^{ox} (right) recorded at 80 K. Fitting parameters are reported in **Supplemental Table 3**.

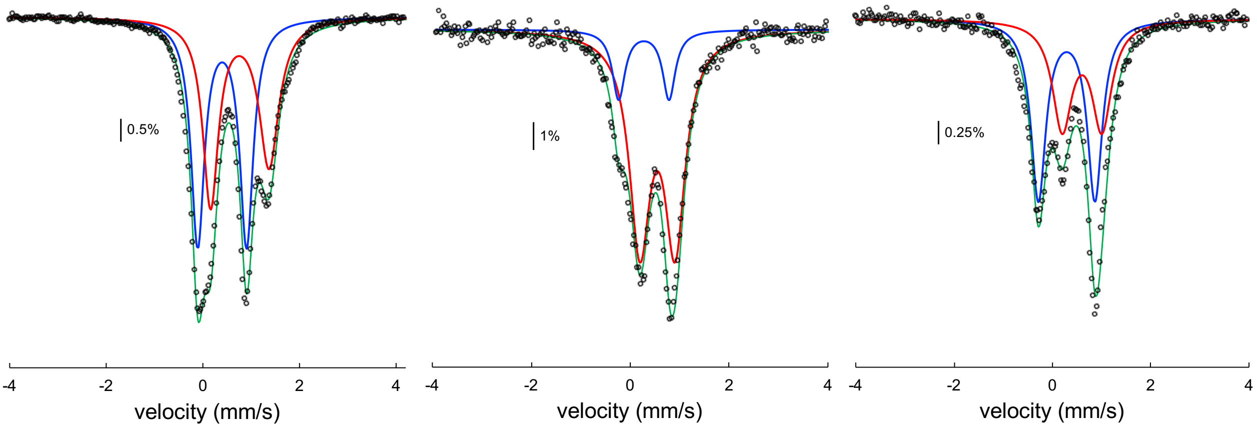

Figure 13. Variable temperature zero-field Mössbauer spectra of crystalline complex **1-[Fe_2S_2]$^+$** recorded at 80 K (left), 140 K (centre), and 180 K (right). The data is fit using two overlapping quadrupole doublets as follows. At 80K, δ_1 = 0.40 mm/s, $|\Delta E_Q|_1$ = 1.01 mm/s (blue) and , δ_2 = 0.76 mm/s, $|\Delta E_Q|_2$ = 1.21 mm/s (red) in a 49:51 ratio. At 140K, δ_1 = 0.28 mm/s, $|\Delta E_Q|_1$ = 1.02 mm/s (blue) and , δ_2 = 0.55 mm/s, $|\Delta E_Q|_2$ = 0.71 mm/s (red) in a 17:83 ratio. At 180 K, δ_1 = 0.30 mm/s, $|\Delta E_Q|_1$ = 1.15 mm/s (blue) and , δ_2 = 0.61 mm/s, $|\Delta E_Q|_2$ = 0.79 mm/s (red) in a 62:38 ratio.

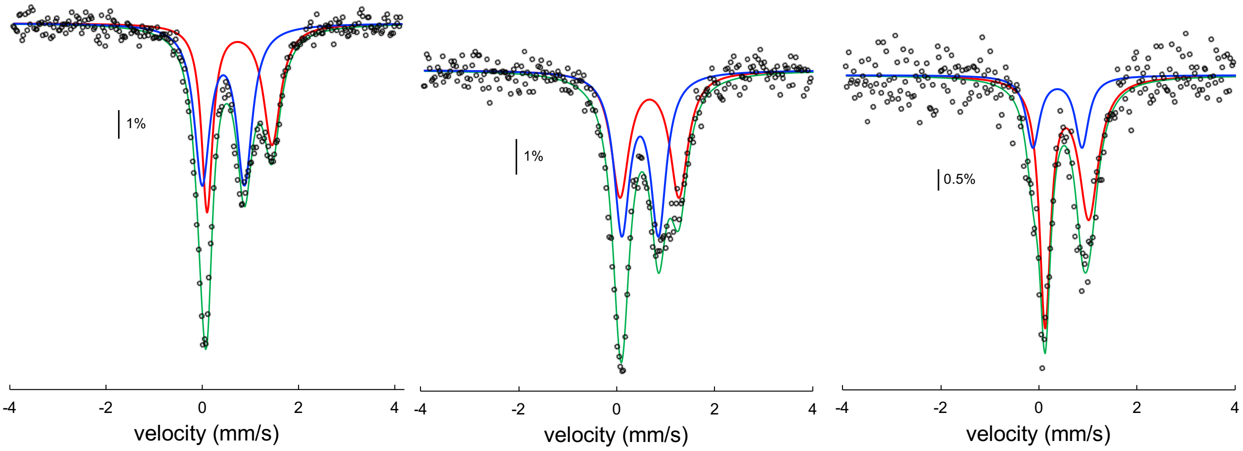

Figure 14. Variable temperature zero-field Mössbauer spectra of crystalline complex **2-[Fe_2Se_2]$^+$** recorded at 80 K (left), 140 K (centre), and 180 K (right). The data is fit using two overlapping quadrupole doublets as follows. At 80K, δ_1 = 0.44 mm/s, $|\Delta E_Q|_1$ = 0.88 mm/s (blue) and , δ_2 = 0.78 mm/s, $|\Delta E_Q|_2$ = 1.35 mm/s (red) in a 54:46 ratio. At 140 K, δ_1 = 0.48 mm/s, $|\Delta E_Q|_1$ = 0.75 mm/s (blue) and , δ_2 = 0.67 mm/s, $|\Delta E_Q|_2$ = 1.20 mm/s (red) in a 53:47 ratio. At 180 K, δ_1 = 0.38 mm/s, $|\Delta E_Q|_1$ = 1.00 mm/s (blue) and , δ_2 = 0.57 mm/s, $|\Delta E_Q|_2$ = 0.89 mm/s (red) in a 26:74 ratio.

Variable Temperature Zero-field Mössbauer Discussion

At 80 K, the Mössbauer spectrum of **1** reveals two overlapping quadrupole doublets corresponding to the "Fe^{3+}" and "Fe^{2+}" sites, in blue and red, respectively (**Supplemental Fig. 13**). At 140 K, the two doublets of the mixed-valent complex have collapsed into one broader doublet (red), centred roughly at the average of the two isomer shifts of the 80 K spectrum with δ_2 = 0.55 mm/s, indicating fast exchange on the Mössbauer timescale (full delocalization). The second doublet (blue, δ_1 = 0.28 mm/s) is assigned as the diferric **1^{ox}**, arising from oxidative decomposition at this temperature, accounting for approximately 20% of the signal. Raising the temperature to 180 K, the amount of decomposition significantly increases to approximately 60% (blue).

At 80 K, the Mössbauer spectrum of **2** reveals two overlapping quadrupole doublets corresponding to the "Fe^{3+}" and "Fe^{2+}" sites, in blue and red, respectively (**Supplemental Fig. 14**). At 140 K, the two doublets of the mixed-valent complex have started to coalesce, but are still fit as two separate doublets with a smaller separation of the isomer shifts ($\delta_2 - \delta_1$ = 0.19 mm/s at 140 K vs. $\delta_2 - \delta_1$ = 0.34 mm/s at 80 K), indicating faster exchange at this temperature. At 180K, the two doublets have collapsed into a single asymmetric quadrupole doublet (red) with δ_2 = 0.57 mm/s. The asymmetric shape has previously been discussed.[2-4] The second doublet (blue, δ_1 = 0.38 mm/s) at 180 K is assigned as the diferric **2^{ox}**, arising from oxidative decomposition at this temperature, accounting for approximately 25% of the signal. The 180 K spectrum in **Supplemental Fig. 14** was measured over 6 hours. Longer measurement times reveal further conversion to **2^{ox}**, with complete oxidation observed after 24 hours. After measuring for 48hours at 180 K, subsequent measurement at 80 K confirmed complete oxidation to **2^{ox}**.

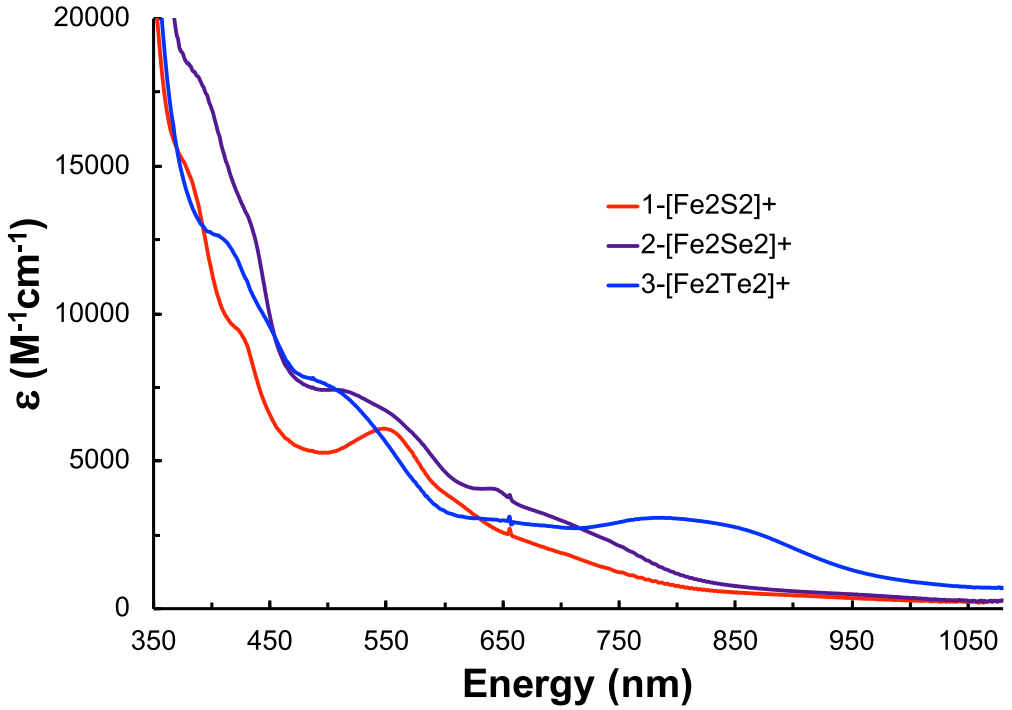

Figure 15. UV-vis spectra of complexes **1** – **3** in THF recorded at 294 K: complex **1** (red), complex **2** (purple), complex **3** (blue).

Inter-valence Charge Transfer Discussion

Robin-Day Class II and III clusters exhibit different absorption properties, with Class II exhibiting weaker spin-independent bands, while Class III yields more intense spin-dependent features.[5] The absorption spectra of **1** and **2** are fairly similar and do not exhibit distinct inter-valence charge-transfer (IVCT) bands in the near-IR (**Supplemental Fig. 15**). In contrast, the absorption spectrum of **3** demonstrates a broad feature centred at ~825 nm (~12100 cm^{-1}) that could potentially be assigned as IVCT, supportive of Class III delocalization. However, for the fully delocalized S=3/2 ground state, the IVCT transition equates to the symmetric to antisymmetric spin-state transition at an energy of 4B. Assignment of this feature at ~12100 cm^{-1} to the IVCT band would imply B=3000 cm^{-1}, an improbably large value (compare to the IVCT-derived B=1350 cm^{-1} of the S=9/2 $[(Me_3tacn)Fe_2(OH)_3]^{2+}$ complex),[6] even considering the effects of vibronic coupling[7] (which are estimated to be smaller in **3** than in the $[(Me_3tacn)_2Fe_2(OH)_3]^{2+}$ complex, **Supplemental Table 11**). While there remains no consensus within the literature on assignments of the IVCT bands for $[Fe_2S_2]^+$ and related complexes (proposals of 530 nm[8], 1070 nm[9], and 5714 nm[2] have been made), an alternative assignment of the 825 nm band in **3** as ligand-to-metal charge transfer is also plausible.

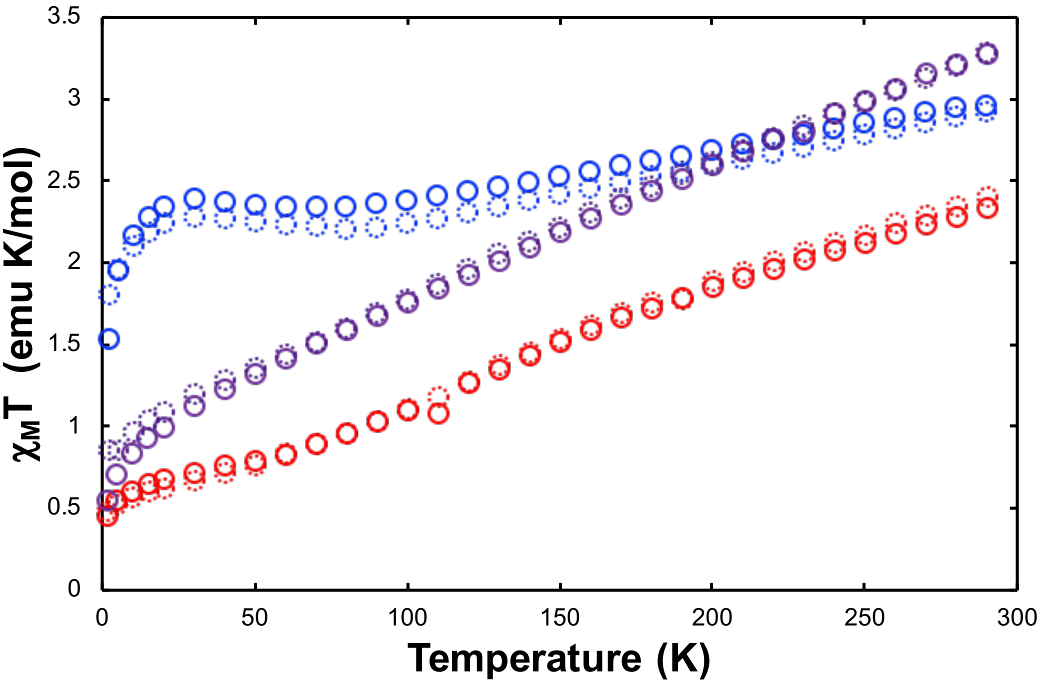

Figure 16. SQUID magnetic susceptibility measurements of crystalline solid samples of **1-3** measured in a field of 1 T (solid circles) and 0.1 T (dashed circles): complex **1-[Fe$_2$S$_2$]$^+$** (red), complex **2-[Fe$_2$Se$_2$]$^+$** (purple), complex **3-[Fe$_2$Te$_2$]$^+$** (blue). The minimal differences between the measurements at 1T and 0.1T demonstrate the absence of any magnetic domain impurities (such as iron oxide nanoparticles). Such impurities would contribute a constant magnetization response regardless of applied field, and thus the 0.1T measurement would result in a 10-fold higher $\chi_M T$ response for these impurities relative to the 1.0T measurement (due to the constant impurity response being divided by a 10-fold smaller applied magnetic field). Therefore, the near linear rise with temperature is not due to a magnetic impurity, but a response of the sample itself and characteristic of the presence of the double-exchange interaction.

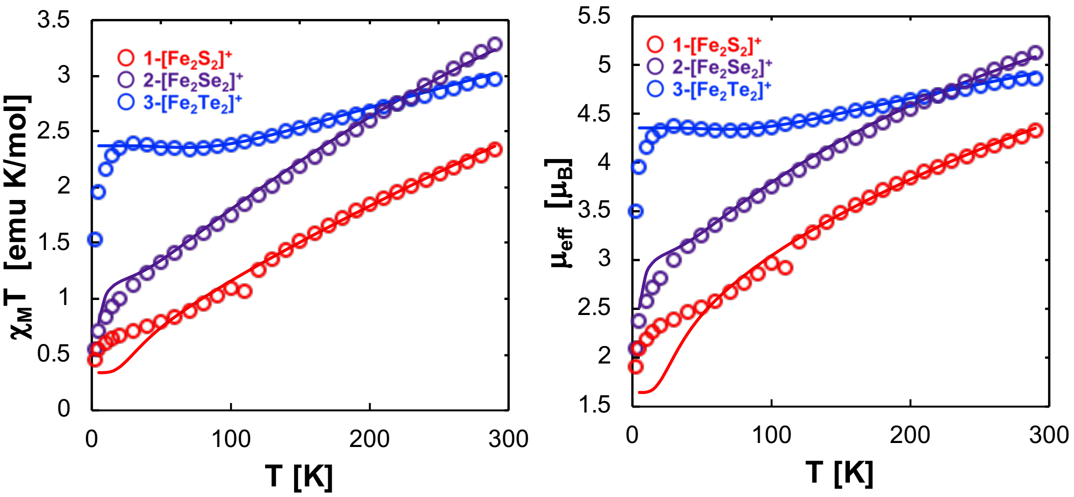

Figure 17. SQUID magnetic susceptibility measurements of crystalline solid samples of 1-3 collected at 1 T plotted as χ_MT vs. T (left) or μ_{eff} vs. T (right). Standard deviation for experimental data is within the data points (open circles). Higher temperature (≥50 K) region is fit to the solved Bleaney-Bowers equation (solid lines) for a high-spin d^5-d^6 mixed-valent centre with the incorporation of double exchange coupling as shown in the equations below. The fits shown are solutions with $|B/J|$ ratios of 2.0 ± 0.2, 2.9 ± 0.2, and 3.7 ± 0.1 for 1, 2, and 3, respectively. See **Supplemental Table 4** for specific g, *J*, and B values. **See below for discussion of limitations of the HDE model to fit the magnetic data.**

$$E_{\pm} = -JS(S+1) \pm B(S+1/2)$$

$$\chi_M = \frac{Ng^2\mu_B^2}{4kT} \cdot \frac{e^{4y}(e^{2y}+1) + 10e^{3x}(e^{7y}+e^{3y}) + 35e^{8x}(e^{8y}+e^{2y}) + 84e^{15x}(e^{9y}+e^{y}) + 165e^{24x}(e^{10y}+1)}{e^{4y}(e^{2y}+1) + 2e^{3x}(e^{7y}+e^{3y}) + 3e^{8x}(e^{8y}+e^{2y}) + 4e^{15x}(e^{9y}+e^{y}) + 5e^{24x}(e^{10y}+1)}$$

$$x = -\frac{J}{kT}; y = -\frac{B}{kT}$$

Discussion of Limitations of the HDE Model to Magnetic Data Fitting

The Bleaney-Bowers equation (above) derived from the Heisenberg double-exchange (HDE) model does not include zero-field splitting, an effect that typically substantially influences the low temperature response of variable temperature magnetic susceptibility measurements. We therefore partially attribute deviations from the fits in this region for the magnetic susceptibility data to the absence of zero-field splitting in the HDE model. Concordantly, the zero-field splittings have been estimated by variable temperate EPR and magnetic Mössbauer studies for complexes **2** and **3**, (see manuscript for discussion).

The HDE model as applied to even 'typical' $[Fe_2S_2]^+$ clusters is still an oversimplification, failing to accurately capture the complex density of states in the low-energy spectrum, as has been previously discussed.[10] Noodleman and coworkers have noted the difficulties of fitting the low-temperature magnetic data for $[Fe_4S_4]^{3+}$cubanes without overparameterizing the solution.[11] Others have chosen to only qualitatively interpret magnetic susceptibility data of FeS clusters out of caution to not overfit the HDE model.[12] Additionally, the simple HDE model fails to account for other effects such as vibronic coupling, which can also significantly perturb the spin ladder. However, from our semi-empirical vibronic coupling analysis (see manuscript), the incorporation of the heavier congeners Se and Te in our iron chalcogenide dimers significantly reduces the localizing effect of the PKS vibration, suggesting the omission of vibronic coupling in the HDE model approaches a reasonable approximation for complexes **2** and **3**. Given the noted shortcomings of the HDE model, particularly at low temperature, we have focused our fitting to $T \geq 50$ K, similar to a previous study of a double-exchange dominated vanadium dimer system.[7] Thus, deviations from the fit at low temperatures are unsurprising given the shortcomings of the HDE model and the especially small J-couplings (resulting in quite condensed spin manifolds) of the present complexes.

Low-temperature deviations notwithstanding (most notably in complex **1**), we believe there is still value in the HDE formalism as presented, particularly in extracting a robust estimate of the ratio $|B/J|$, which emphasizes the overall effect of heavier chalcogenide substitution on the electronic exchange in these dimers. Furthermore, the saturation magnetization, EPR, and magnetic Mössbauer data are all consistent with an S=1/2 ground state for **1-[Fe$_2$S$_2$]$^+$**, indicating the low-temperature deviation from the SQUID fit for this complex neither contradicts nor detracts from our overall analysis.

Table 4. Parameters for SQUID fits of complexes **1**, **2**, and **3**.

	g	J (cm^{-1})^a	B (cm^{-1})^a	$\|B/J\|^a$
**1-[Fe$_2$S$_2$]$^+$**	1.90^b	-55 ± 5	110 ± 20	2.0 ± 0.2
**2-[Fe$_2$Se$_2$]$^+$**	1.92^b	-50 ± 5	145 ± 25	2.9 ± 0.2
**3-[Fe$_2$Te$_2$]$^+$**	2.25^c	-200 ± 25	750 ±110	3.7 ± 0.1

[a]Multiple reasonable fits could be generated using varying *J*, *B* values. The multiple fits generated demonstrate that while no unique solution of *J* and *B* may be determined, *J* and *B* appear well correlated, resulting in a robust measure of their ratio |*B*/*J*|. Average values of *J*, *B*, and |*B*/*J*| are reported with the standard deviation from the multiple fits (determined from visual inspection). Examples of alternative fits are shown in **Figures S3-5**. [b]g_{iso} values taken from S=1/2 signals measured by EPR. [c]Estimated from saturation magnetization data.

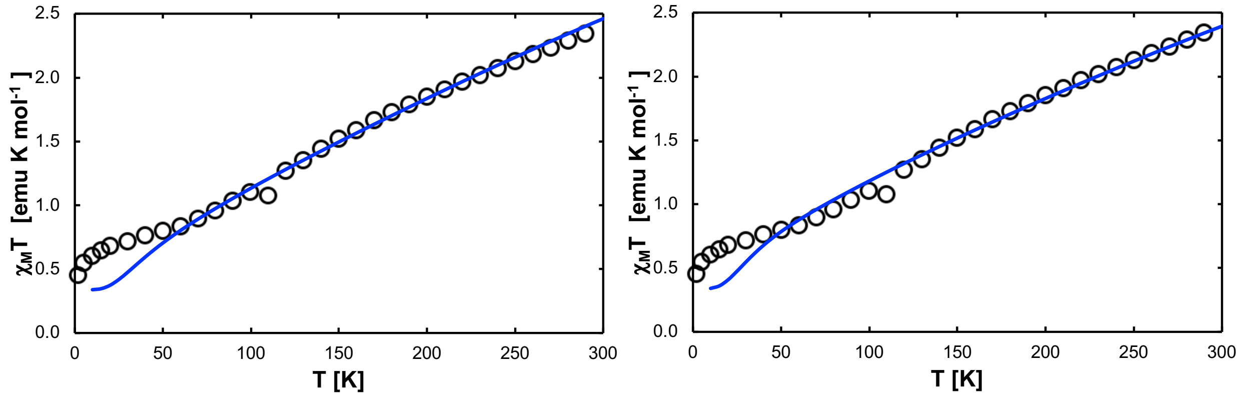

Figure 18. Representative alternative fits of **1-[Fe_2S_2]$^+$** SQUID data. Experimental data shown as open circles, fits shown as solid blue lines. Fit with g=1.90, *J*=-50 cm^{-1}, *B*=90 cm^{-1} (left), and g=1.90, *J*=-60 cm^{-1}, *B*=130 cm^{-1} (right).

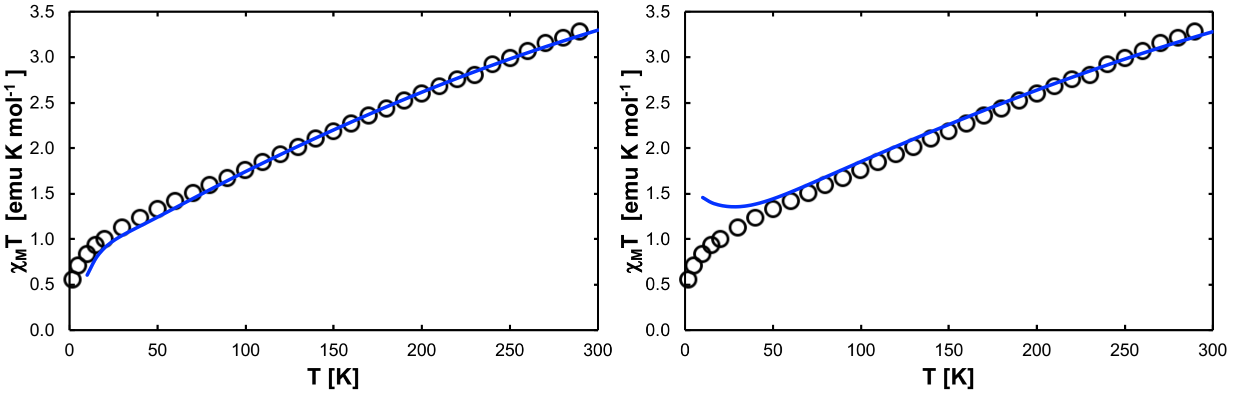

Figure 19. Representative alternative fits of **2-[Fe_2Se_2]$^+$** SQUID data. Experimental data shown as open circles, fits shown as solid blue lines. Fit with g=1.92, *J*=-45 cm^{-1}, *B*=120 cm^{-1} (left), and g=1.90, *J*=-55 cm^{-1}, *B*=170 cm^{-1} (right).

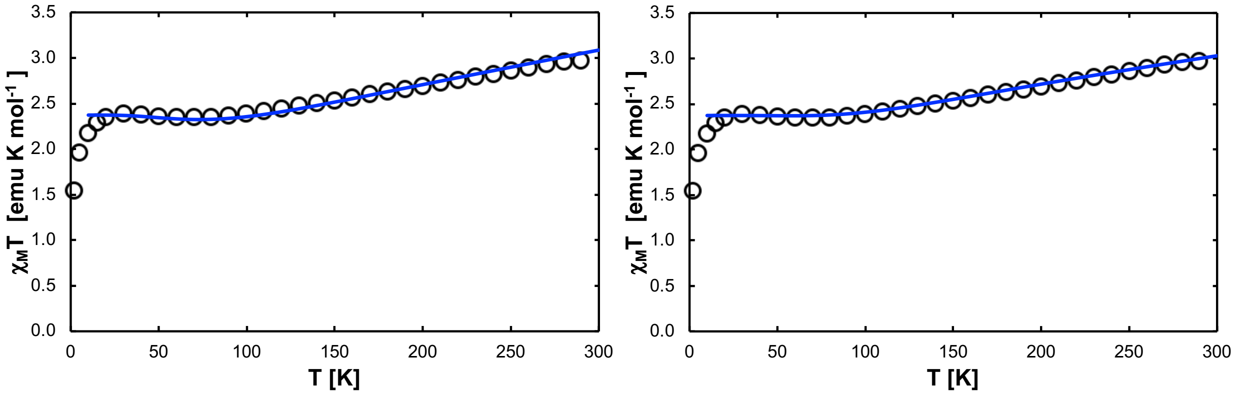

Figure 20. Representative alternative fits of **3-[Fe_2Te_2]$^+$** SQUID data. Experimental data shown as open circles, fits shown as solid blue lines. Fit with g=2.25, *J*=-175 cm^{-1}, *B*=640 cm^{-1} (left), and g=2.25, *J*=-225 cm^{-1}, *B*=860 cm^{-1} (right).

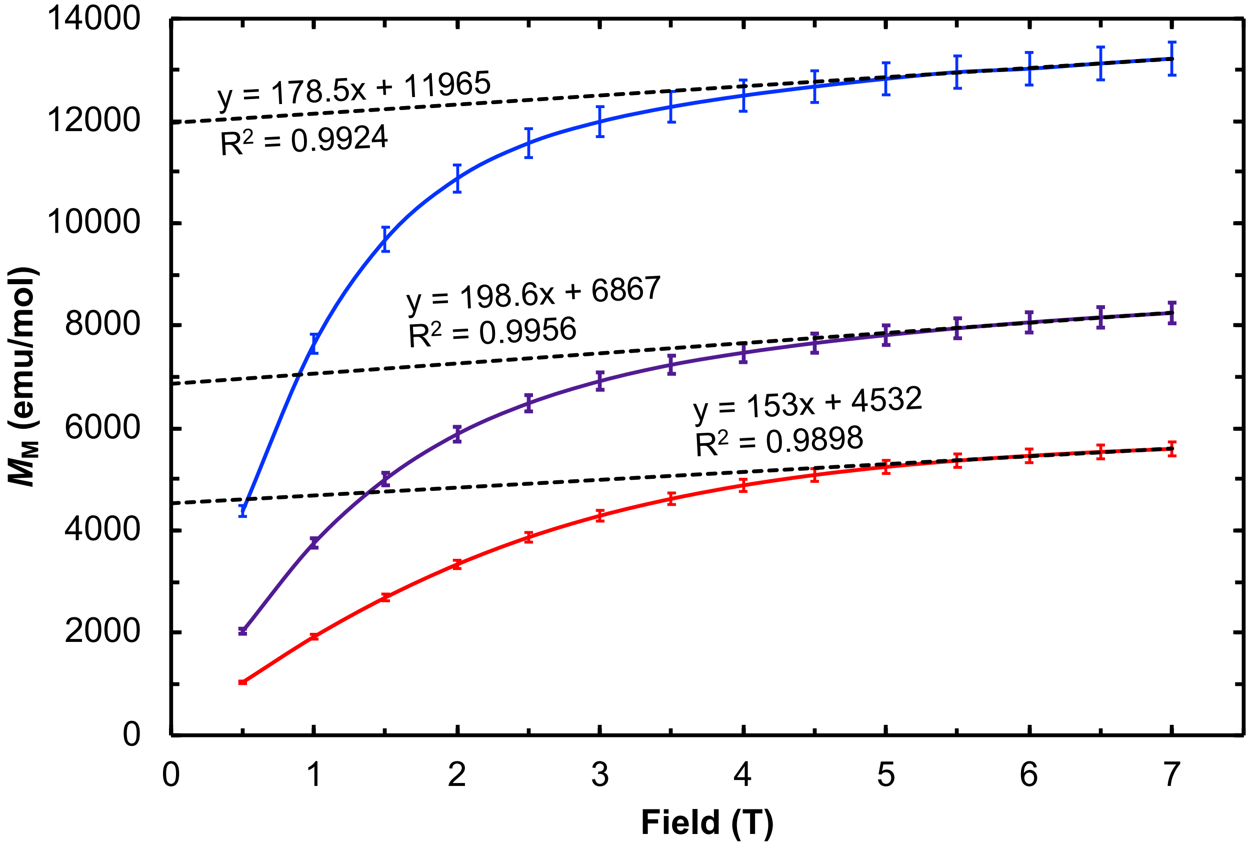

Figure 21. Saturation magnetization data recorded at 2K for complexes **1-[Fe_2S_2]$^+$** (red), **2-[Fe_2Se_2]$^+$** (purple), and **3-[Fe_2Te_2]$^+$** (blue). Experimental data points are shown by their vertical error bars and connected by solid lines to guide the eye. The linear response at higher field (>5 T) is indicative of magnetic saturation. The data under saturating conditions (5.5 ≤ T ≤ 7) were fit to the expression $M_M = M_S + xH$, where M_M is the molecular magnetization, M_S is the saturation magnetization, x is a constant and H is the applied field. The fits are given as dashed lines with corresponding R^2 values. The resulting M_S values were then converted to Bohr magnetons, yielding values of 0.81 μ_B, 1.23 μ_B, and 2.14 μ_B for **1**, **2**, and **3**, respectively. Accounting for random orientation of the crystallites within the sample powder, the magnetic moment along the main magnetization axis at saturation was calculated to be 1.6±0.1 μ_B, 2.5±0.1 μ_B, and 4.3±0.1 μ_B per molecule for complexes **1**, **2**, and **3**, respectively.

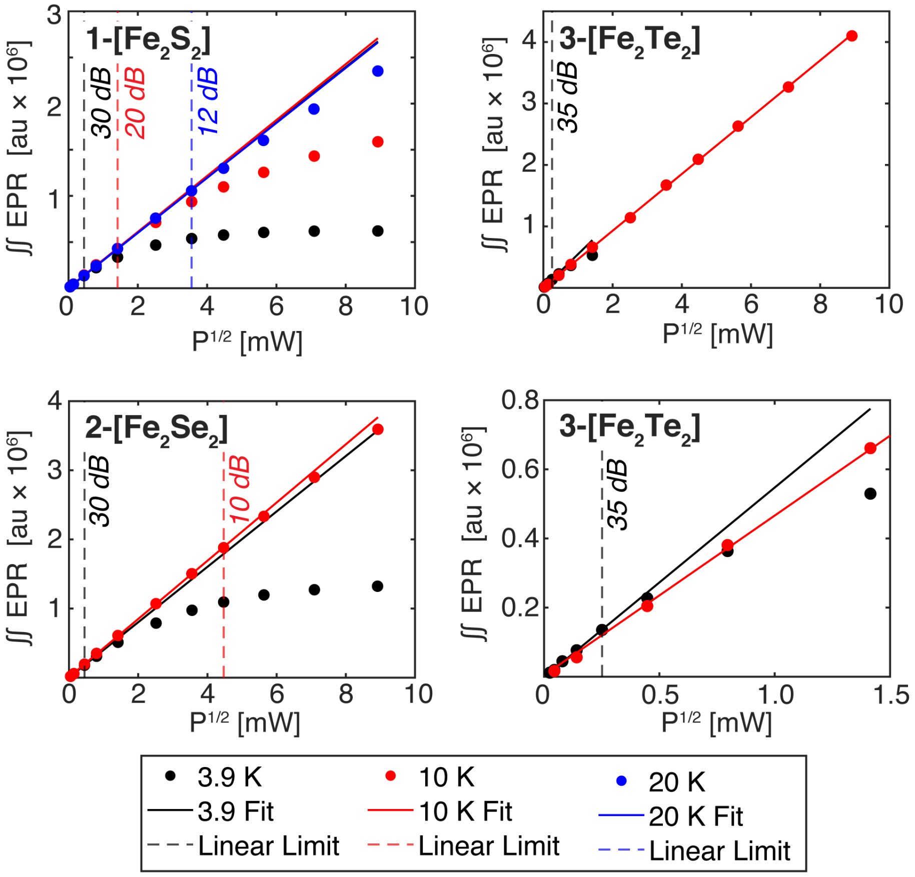

Figure 22. EPR power saturation curves determined from the temperature normalized double integration of the as-collected derivative EPR signals versus the square-root of the applied microwave power at fixed temperatures of 3.9, 10 and 20 K. The solid lines are best-fits of the linear response regions of the power-saturation curve where the vertical dashed line represented the upper limit of the linear region. The microwave source's unattenuated power is 200 mW.

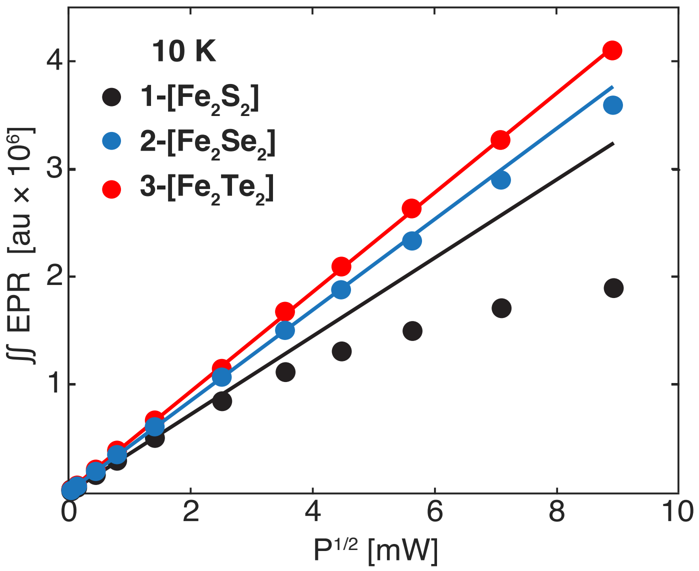

Figure 23. Comparison of EPR power saturation curves of **1-3** measured at 10 K. The linear lines represent the regions linear response and unsaturated signal.

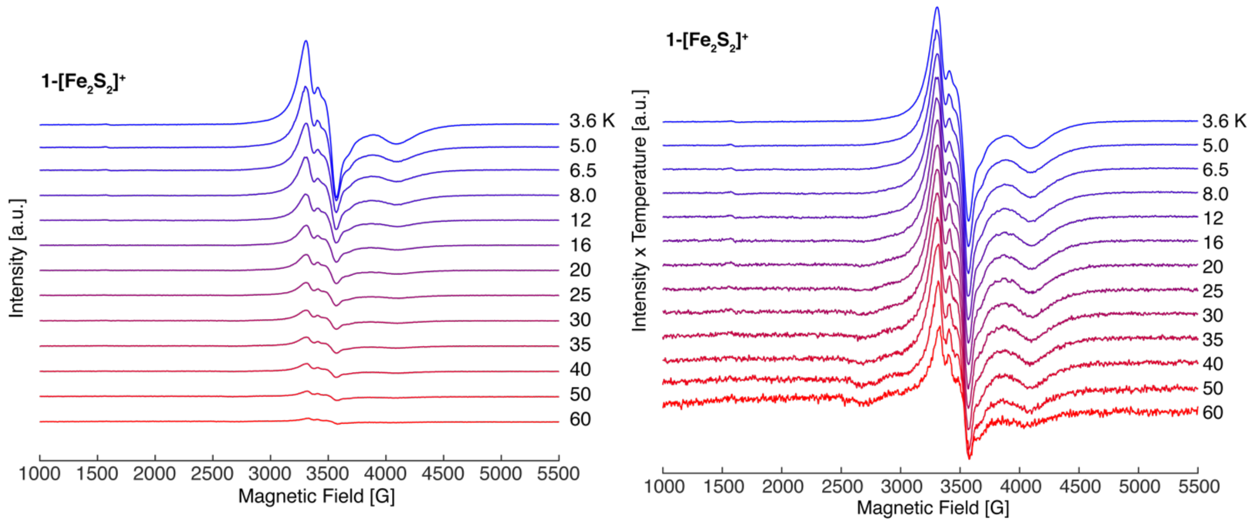

Figure 24. VT X-band CW-EPR of **1** collected under non-saturating microwave power conditions and as collected (left) and intensity scaled (right) by the sample temperature (signal x T [K]). Parameters: 81.92 ms time constant; 81.92 ms conversion time; 4096 points; 100 kHz field modulation; 6 G modulation amplitude; 9.636 GHz microwave frequency.

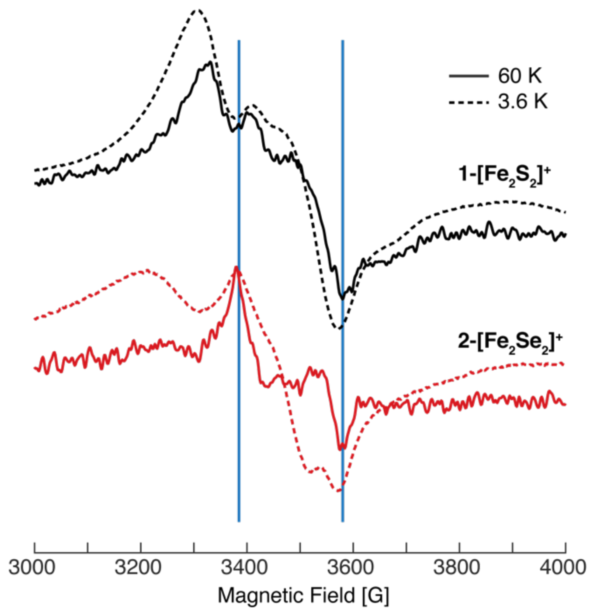

Figure 25. EPR comparison of **1** and **2** at low (3.6 K) and warmer (60 K) temperatures, showing the presence of the *S*=1/2 impurity marked by the vertical lines.

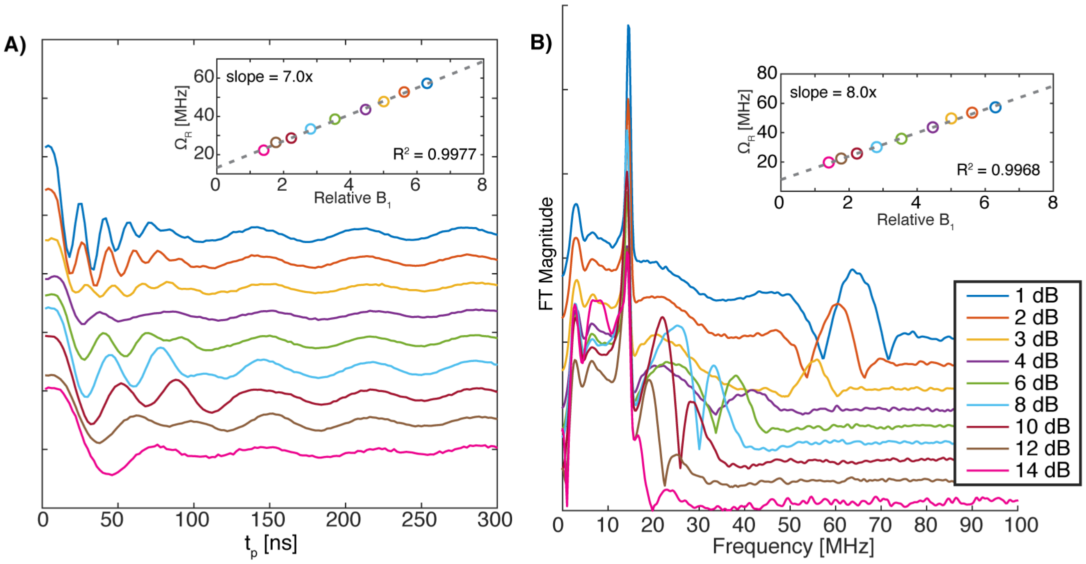

Figure 26. (**A**) Variable microwave power nutation time traces of **1** at 3400 G and 5.5 K and the respective Fourier transform (**B**). The nutation frequency at each microwave power in **A** is determined by the minimum of the first oscillation. Oscillations >200 ns corresponds to the ^{1}H Larmor response, demonstrating the short phase-memory of the spin-nutation. The inset plot exhibits a linear relationship of relative microwave power, B_1, and nutation frequency. The relative microwave power is determined by $B_1 = \sqrt{\frac{10^{-0.1A}}{10^{-1.7}}}$, where A is the microwave attenuation in units of dB. As both plots of nutation frequency vs B_1 exhibit similar slopes, determination of the nutation frequency as performed in **A** is reliable.

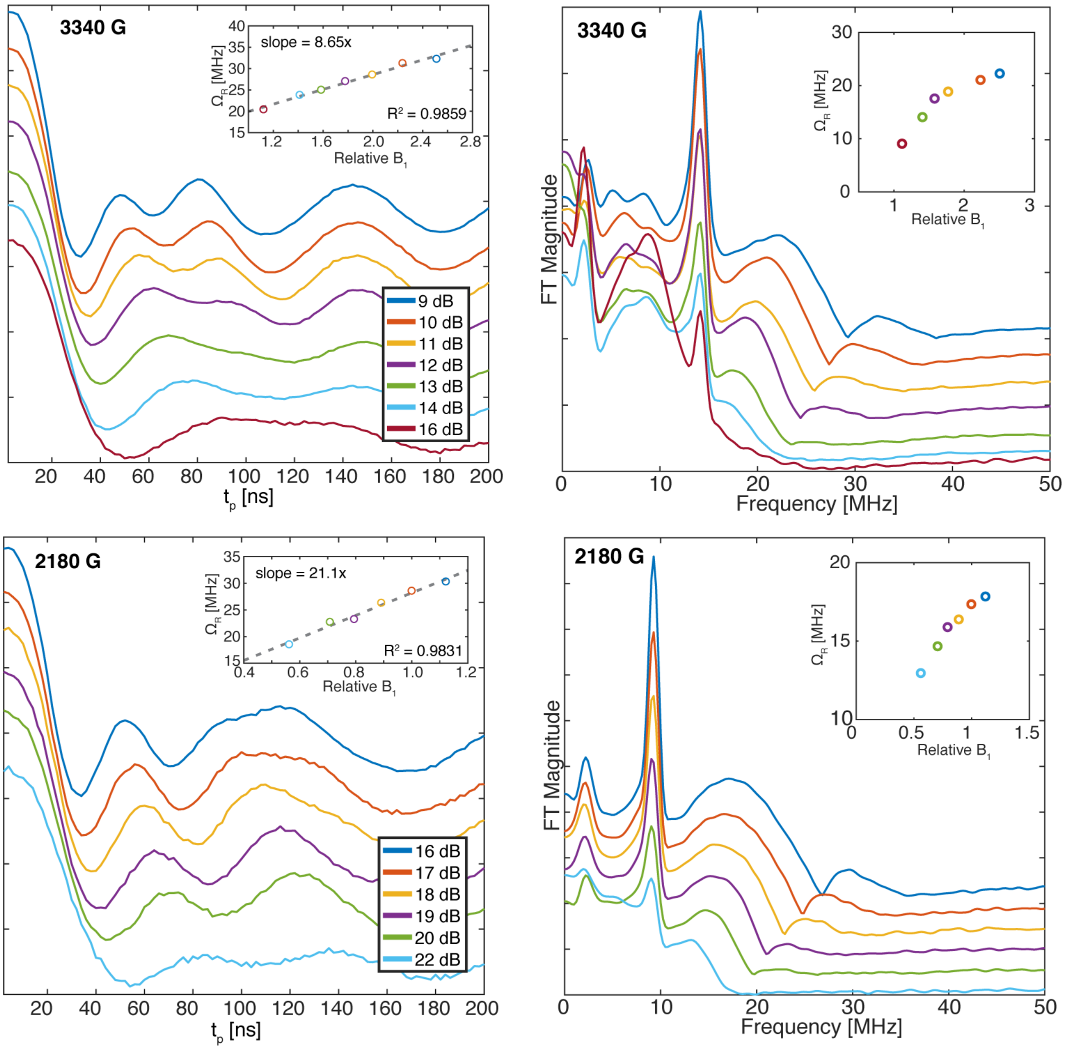

Figure 27. Variable microwave power nutation traces of **2** at 3340 G (top) and 2180 G (bottom) and 5.5 K. The time domain traces for each experiment are displayed, left, where the nutation frequency at each microwave power is determined by the minimum of the first oscillation. Oscillations >200 ns corresponds to the ^{1}H Larmor response. The sharp peaks at ~14.2 MHz (3340 G) and ~9.3 MHz (2180 G) observed at all microwave powers corresponds to the ^{1}H Larmor frequency. At 3340 G, a linear trend of B_1 vs nutation frequency is observed, with a similar slope compared to **1**, confirming the similar S = 1/2 identity of the observed EPR resonance. The Fourier Transform of the data, right, exhibits approximately the same nutation frequencies as determined from the minimum of the first oscillations at higher microwave powers. Constructive interreference effects of the nutation waveform and the ^{1}H waveform make it difficult to accurately determine nutation frequencies at lower microwave powers. At 2180 G, a linear trend of B_1 vs nutation frequency is also observed, with an increased slope compared to **2** at 3340 G by a factor of 2.43. The anticipated ratio is $[S(S+1)]^{1/2}/[S'(S'+1)]^{1/2} \approx 2.24$, confirming the similar S = 3/2 identity of the observed EPR resonance.

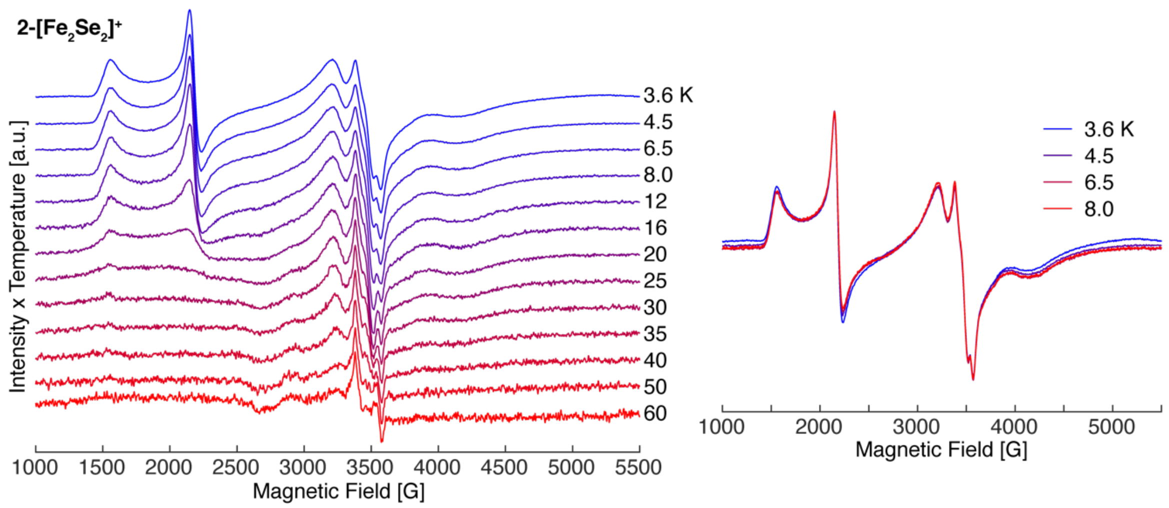

Figure 28. VT X-band CW-EPR of **2** collected under non-saturating microwave power conditions and scaled by the sample temperature (signal x T [K]) and overlays of 3.6 – 8K (right). Parameters: 81.92 ms time constant; 81.92 ms conversion time; 4096 points; 100 kHz field modulation; 6 G modulation amplitude; 9.637 GHz microwave frequency.

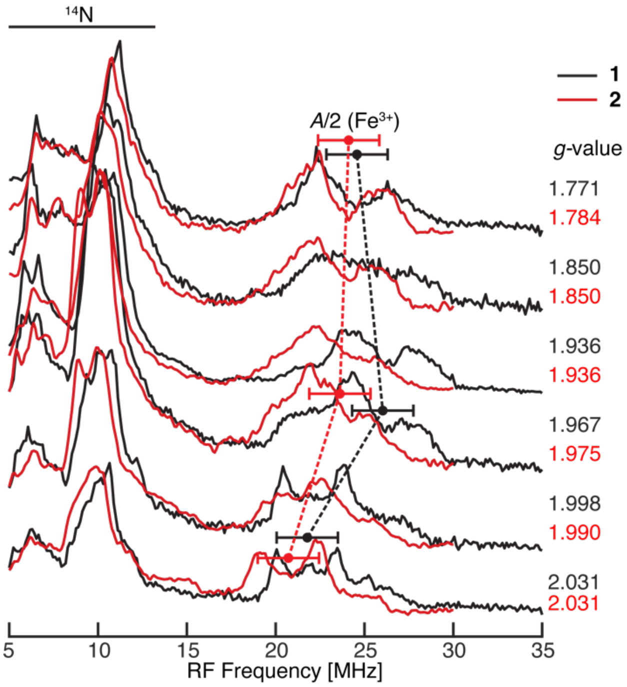

Figure 29. Q-band 2 K Davies ENDOR of **1** (black) and **2** (red) with $^{57}Fe^{3+}$ responses at higher frequency and predominantly ^{14}N response at lower frequencies. For the single Fe ion hfc observed in the ENDOR pattern for **1** and **2**, the hfc is designated by goal posts separated by $2\times\nu_n(^{57}Fe) \sim 3.4$ MHz and centred at $A/2$. In the classic iron hyperfine spin projection scheme for an antiferromagnetically coupled d^5-d^6 centre, $\mathbf{A} = -7/3\mathbf{A}(Fe^{3+}) + 4/3\mathbf{A}(Fe^{2+})$, only the larger Fe^{3+} hfc is observed. Experimental parameters: 35.05 GHz microwave frequency, $\pi/2 = 60$ ns, $\tau = 600$ ns, $T_{RF} = 35$ μs, 50 ms repetition rate.

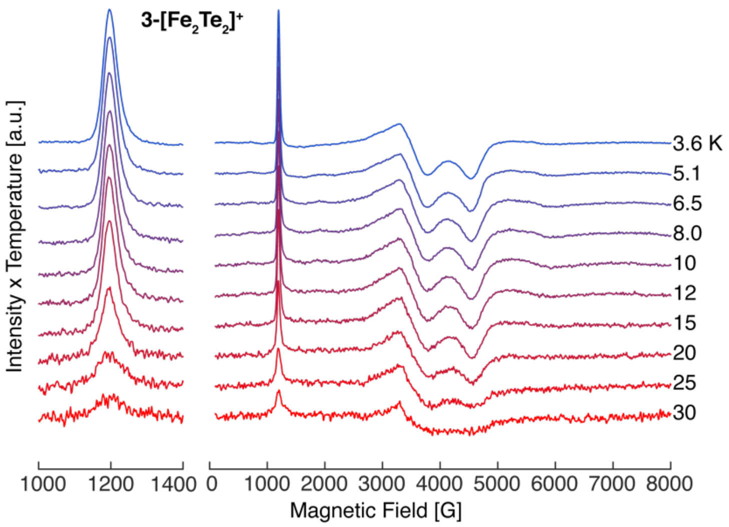

Figure 30. VT X-band CW-EPR of **3** collected under non-saturating microwave power conditions and scaled by the sample temperature (signal x T [K]). Parameters: 81.92 ms time constant; 81.92 ms conversion time; 4096 points; 100 kHz field modulation; 6 G modulation amplitude; 9.637 GHz microwave frequency.

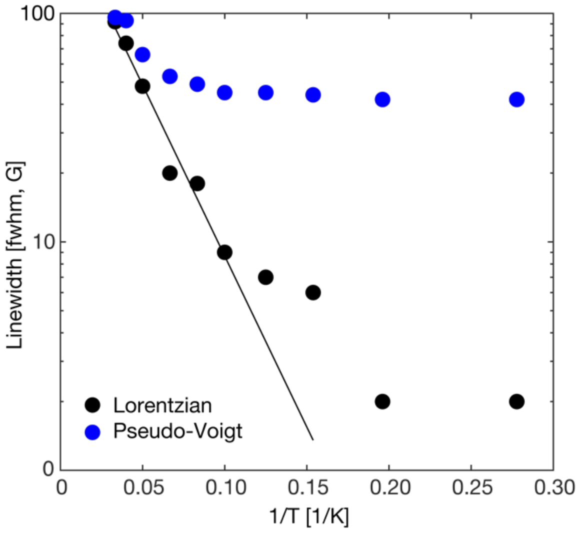

Figure 31. Fitted pseudo-Voigt linewidth to the g_1 (~1200 G) feature of **3**. At temperatures <10 K, no overall broadening of the g_1 feature occurs (blue dots). At higher temperatures, >10 K, an increased Lorentzian contribution begins to dominate the line shape of g_1. The effective Lorentzian linewidth is proportional to the energy separation of low-lying excited states.[13] In the case of **3**, the higher energy doublet of the ZFS is populated at higher temperatures. Additionally, the linewidth here is also inversely proportional to the spin-lattice relaxation rate (T_1), all given by:

$$\delta \propto T_1^{-1} \propto A(e^{\Delta/kT} - 1)^{-1}$$

where, δ is the Lorentzian linewidth, and Δ/kT is the Boltzmann distribution. From the fitted line of the linear response of the Lorentzian line component, the slope is $|\Delta|$~15 cm^{-1}, yielding a very rough estimate of the ZFS, |D| ~ 15 cm^{-1}. This is in approximate agreement with the fitted D = 11 cm^{-1} from Mössbauer spectroscopy, **Supplemental Fig. 33**.

Table 5. Parameters for magnetic Mössbauer simulations (**Supplemental Figs. 32-34**). All simulations employ linewidths of Γ=0.5 mm/s.

		S=1/2		*S*=3/2
		"Fe^{3+}"	"Fe^{2+}"	
**1-[Fe_2S_2]$^+$**	δ^a (mm/s)	0.44	0.74	
	$\Delta E_Q{}^b$ (mm/s)	-1.04	1.88	
	η^c	1.2	0	
	A_{xx} (T)	-34	-5	
	A_{yy} (T)	-31	16	
	A_{zz} (T)	-39	13	
	g_x, g_y, g_z	2.09, 1.96, 1.67		
**2-[Fe_2Se_2]$^+$**	δ (mm/s)	0.44	0.78	0.56
	ΔE_Q (mm/s)	-0.84	1.72	0.9
	η	1	0	0
	A_{xx} (T)	-30	-4	-10
	A_{yy} (T)	-30	5	-10
	A_{zz} (T)	-30	12	-10
	g_x, g_y, g_z	2.15, 1.97, 1.65		4.38, 3.16, 1.94
	D, *E*/*D*			+11, 0.12
**3-[Fe_2Te_2]$^+$**	δ (mm/s)			0.65
	ΔE_Q (mm/s)			1.13
	η			0
	A_{xx} (T)			-10
	A_{yy} (T)			-10
	A_{zz} (T)			-10
	g_x, g_y, g_z			5.74, 1.95, 1.52
	D, *E*/*D*			+11, 0.33

[a]Isomer shift relative to alpha-Fe at room temperature.
[b]Quadrupole splitting with sign of the main component V_{zz} of the electric field gradient.
[c]Asymmetry parameter $\eta = (V_{yy} - V_{xx})/V_{zz}$.

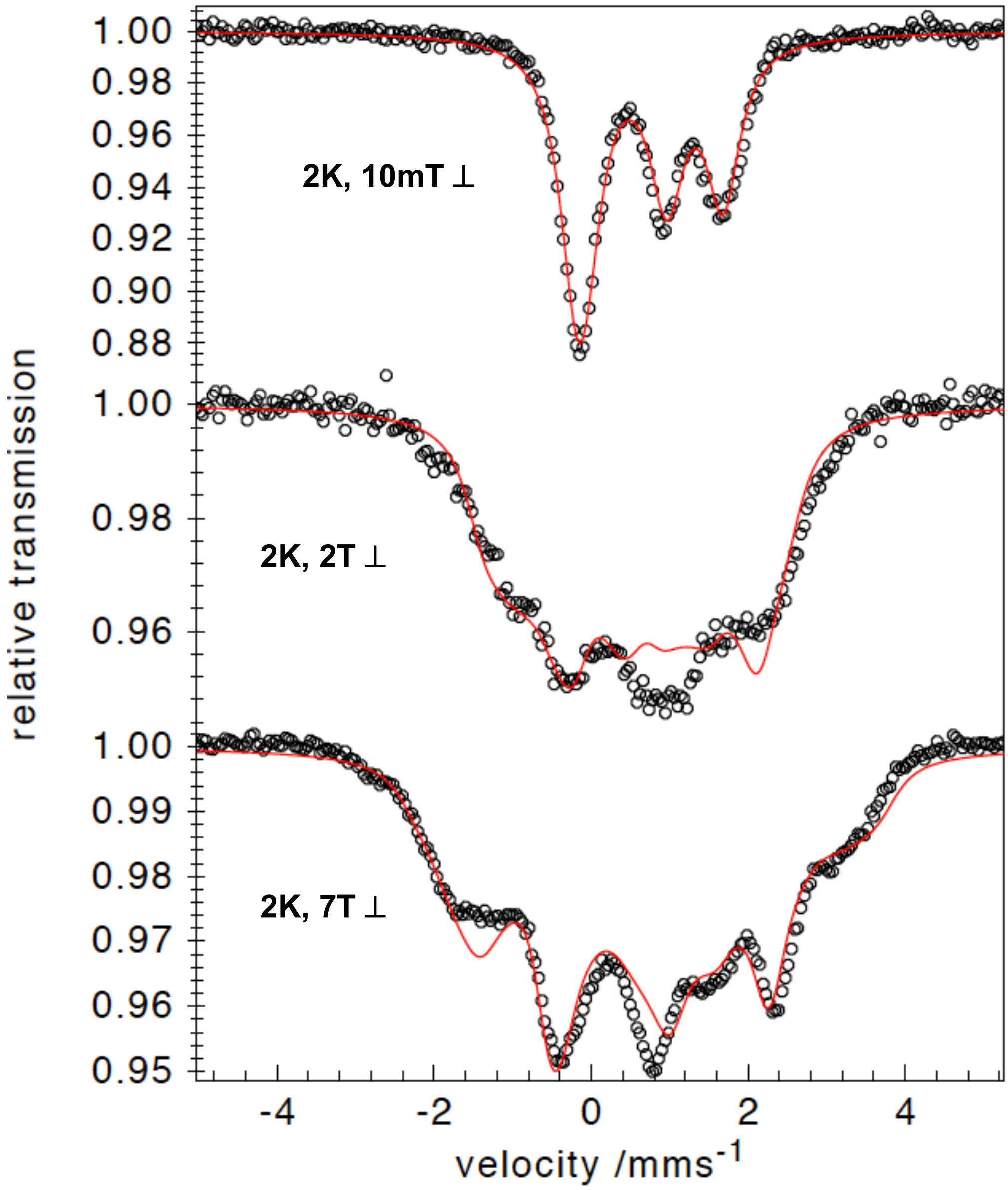

Figure 32. Magnetic Mössbauer measurements of **1** with simulated fittings. See **Supplemental Table 5** for simulation parameters.

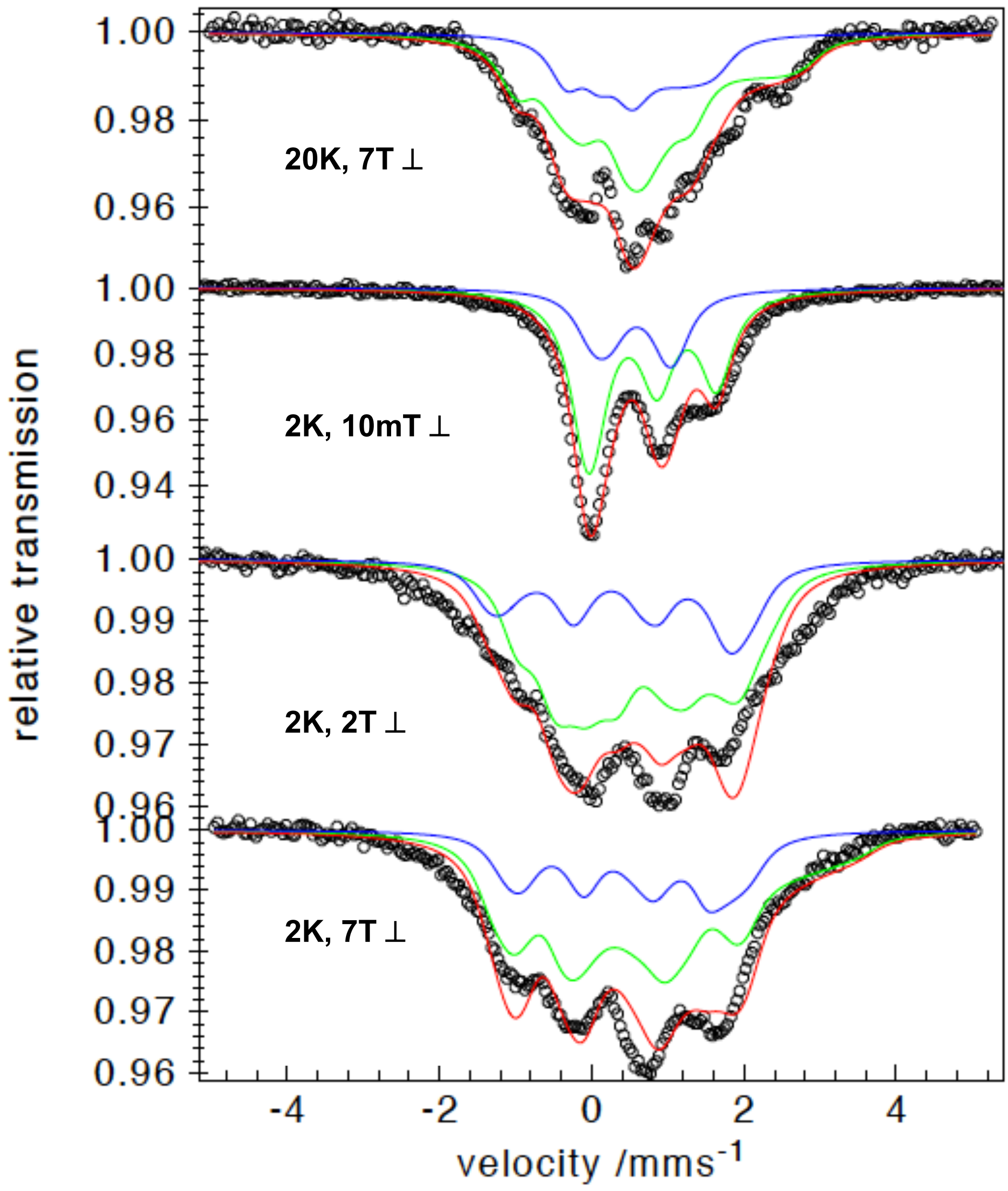

Figure 33. Magnetic Mössbauer measurements of **2** with simulated fittings. The green line corresponds to the S=1/2 simulation (70%), the blue line the S=3/2 simulation (30%), and the red line the sum of the two sub-spectra. See **Supplemental Table 5** for simulation parameters.

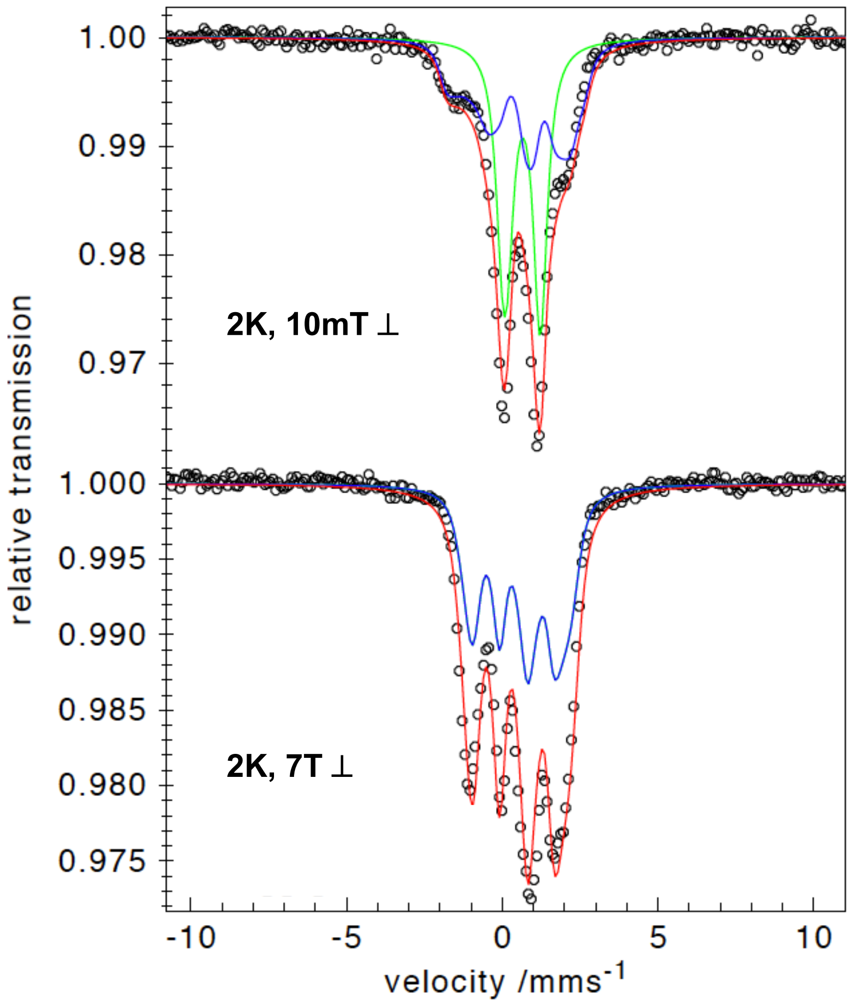

Figure 34. Magnetic Mössbauer measurements of **3** with fittings. See **Supplemental Table 5** for simulation parameters. The magnetic Mössbauer spectrum was fit as a 50:50 mix of two sub-spectra, both with identical parameters (**Supplemental Table 5**). The two sub-spectra differed only in their relaxation behaviour, with one assuming fast relaxation (green line) and the other assuming slow relaxation (blue line). This relaxation subpopulation is interpreted as resulting from the two crystalline forms of **3**, namely **3'** and **3''**, which are approximately in a 1:1 ratio as suggested by powder XRD (**Supplemental Fig. 11**).

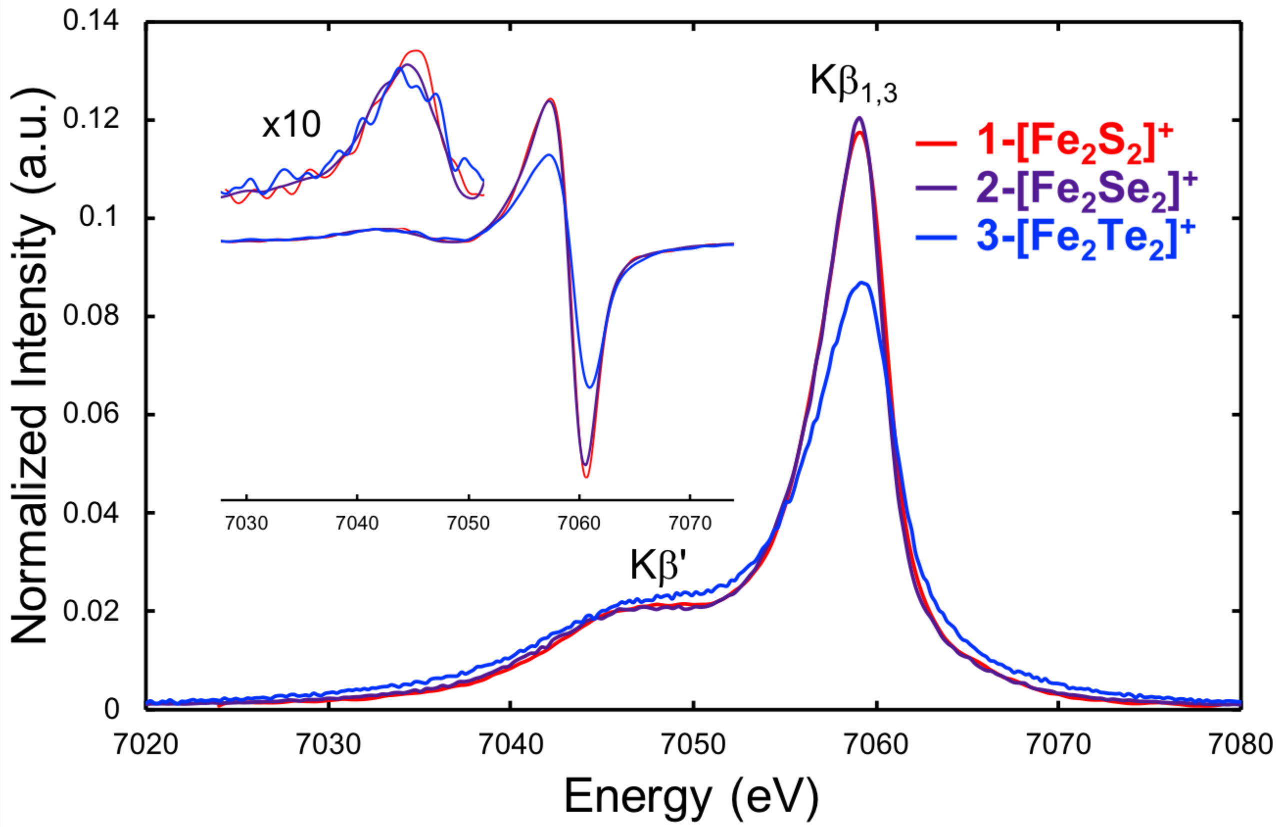

Figure 35. Fe Kβ mainlines of complexes **1-[Fe_2S_2]$^+$** (red), **2-[Fe_2Se_2]$^+$** (purple), and **3-[Fe_2Te_2]$^+$** (blue). Inset shows the first derivative for all three spectra, demonstrating similar splitting of the K$\beta_{1,3}$ and Kβ' features for all three complexes, indicative of similar ligand fields across the series. Thus, all three spectra are consistent with locally high-spin Fe. Data for **1** was adapted with permission from reference [14].

Table 6. Comparison of selected bond metrics of the experimental crystal structure and DFT calculated structures in the HS (S=9/2) and BS (S=1/2) solutions for complexes **1-3**.

	**1-[Fe_2S_2]$^+$**			**2-[Fe_2Se_2]$^+$**			**3-[Fe_2Te_2]$^+$**		
	DFT		Exp.	DFT		Exp.	DFT		Exp.
	HS S=9/2	BS S=1/2		HS S=9/2	BS S=1/2		HS S=9/2	BS S=1/2	
Fe–Fe (Å)	3.048	2.832	2.807	3.180	2.983	2.966	3.358	3.173	3.160
Fe_1–Q (Å)	2.283	2.195		2.409	2.323		2.613	2.535	
Fe_2–Q (Å)	2.283	2.295		2.407	2.444		2.615	2.646	
Fe–Q_{av} (Å)	2.283	2.246	2.238	2.408	2.383	2.364	2.614	2.590	2.572
Fe-Q-Fe (°)	83.8	78.2	77.7	82.6	77.5	77.7	80.0	75.5	75.71
Fe_1–N_1 (Å)	2.051	2.072		2.047	2.061		2.020	2.029	
Fe_1–N_2 (Å)	2.058	2.054		2.037	2.046		2.035	2.046	
Fe_2–N_1 (Å)	2.051	2.088		2.046	2.054		2.021	2.025	
Fe_2–N_2 (Å)	2.058	2.085		2.037	2.048		2.035	2.039	
Fe–N_{avg} (Å)	2.055	2.075	2.099	2.042	2.052	2.071	2.028	2.035	2.062

Table 7. DFT Calculated Mössbauer Isomer Shifts (δ).

		Calculated δ (mm/s)	Experimental δ (mm/s)
[Fe_2Te_2]$^{2+}$ BS	Fe1	0.51	0.42
	Fe2	0.52	0.42
[Fe_2Te_2]$^{2+}$ HS	Fe1	0.54	0.42
	Fe2	0.54	0.42
[Fe_2Te_2]$^+$ BS	Fe1	0.58	0.64
	Fe2	0.88	0.64
[Fe_2Te_2]$^+$ HS	Fe1	0.76	0.64
	Fe2	0.76	0.64
[Fe_2Se_2]$^{2+}$ BS	Fe1	0.41	0.38
	Fe2	0.41	0.38
[Fe_2Se_2]$^{2+}$ HS	Fe1	0.44	0.38
	Fe2	0.44	0.38
[Fe_2Se_2]$^+$ BS	Fe1	0.46	0.44
	Fe2	0.77	0.78
[Fe_2Se_2]$^+$ HS	Fe1	0.64	0.44
	Fe2	0.64	0.78
[Fe_2S_2]$^{2+}$ BS	Fe1	0.39	0.34
	Fe2	0.39	0.34
[Fe_2S_2]$^{2+}$ HS	Fe1	0.42	0.34
	Fe2	0.42	0.34
[Fe_2S_2]$^+$ BS	Fe1	0.42	0.4
	Fe2	0.75	0.76
[Fe_2S_2]$^+$ HS	Fe1	0.64	0.4

Fe2	0.64	0.76

Table 8. Calculated *J*-couplings determined using the Yamaguchi spin-projection method for complexes $\mathbf{1^{ox}}$-$\mathbf{3^{ox}}$ and **1-3**.

	J (cm^{-1})
$\mathbf{1^{ox}}$-$\mathbf{[Fe_2S_2]^{2+}}$	-182
$\mathbf{2^{ox}}$-$\mathbf{[Fe_2Se_2]^{2+}}$	-175
$\mathbf{3^{ox}}$-$\mathbf{[Fe_2Te_2]^{2+}}$	-155
**1**-$\mathbf{[Fe_2S_2]^{+}}$	-53
**2**-$\mathbf{[Fe_2Se_2]^{+}}$	-60
**3**-$\mathbf{[Fe_2Te_2]^{+}}$	-39

Table 9. Mulliken spin population analysis for complexes $\mathbf{1^{ox}}$-$\mathbf{3^{ox}}$ and **1-3**.

	Fe1	Fe2	Q1	Q2	ΣFe	ΣQ
$\mathbf{1^{ox}}$-$\mathbf{[Fe_2S_2]^{2+}}$	3.88	-3.88	0.02	-0.02	0	0
$\mathbf{2^{ox}}$-$\mathbf{[Fe_2Se_2]^{2+}}$	3.86	-3.86	0.02	-0.01	0	0.01
$\mathbf{3^{ox}}$-$\mathbf{[Fe_2Te_2]^{2+}}$	3.78	-3.78	0.01	-0.01	0	0
**1**-$\mathbf{[Fe_2S_2]^{+}}$	3.84	-3.55	0.32	0.28	0.29	0.60
**2**-$\mathbf{[Fe_2Se_2]^{+}}$	3.82	-3.61	0.36	0.31	0.21	0.67
**3**-$\mathbf{[Fe_2Te_2]^{+}}$	3.73	-3.60	0.41	0.36	0.13	0.77

Table 10. Calculated PKS vibration frequencies (cm^{-1}).

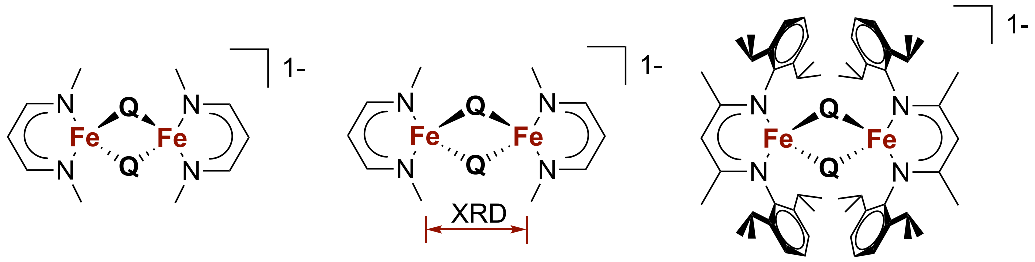

L1 **L2** **L3**

	**L1**^{a}	**L2**	**L3**
$\mathbf{[Fe_2S_2]^{+}}$	285^{b}	286	295
$\mathbf{[Fe_2Se_2]^{+}}$	145	146	160
$\mathbf{[Fe_2Te_2]^{+}}$	119	123	141

^{a}Three different models were employed to explore ligand electronic and steric effects on the PKS vibration. L1 corresponds to a computationally minimal system with Me-substituted α-nitrogen and H-substituted β-carbon β-diketiminate ligand. The L2 corresponds to the L1 ligand with the Fe–Fe distance constrained to the experimentally observed distance from the single crystal XRD structures for **1-3**. Model L3 corresponds to the full experimental β-diketiminate ligand (2,6-diisopropylphenyl-substituted α-nitrogen, Me-substituted β-carbon). All calculations were performed on the BS (S=1/2) solution. ^{b}The PKS vibration was split into two modes (see **Supplemental Fig. 36**) and the average value is reported.

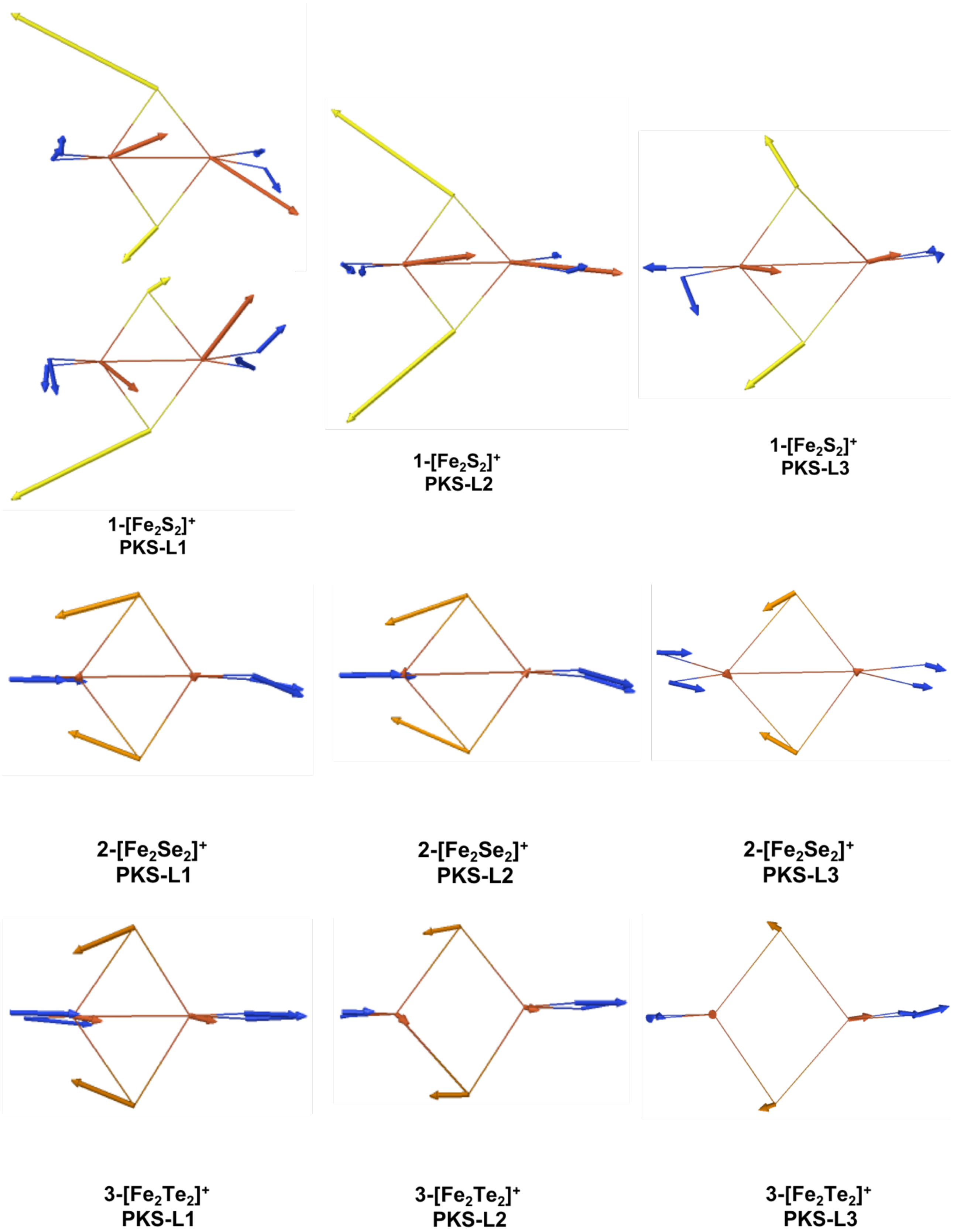

Figure 36. Visualizations of the DFT calculated PKS vibrations for complexes **1** (top), **2** (middle), and **3** (bottom) of L1 (left), L2 (centre), and L3 (right). For clarity, only the $N_4Fe_2Q_2$ core is shown (view

is perpendicular to the Fe_2Q_2 plane). Displacement vectors are represented as arrows; nitrogen in blue, iron in orange, sulphur in yellow, selenium in light orange, and tellurium in burnt orange. Qualitatively, the magnitude of the chalcogenide bridge displacement vector decreases as a function of chalcogenide with S > Se > Te, and similarly the model constraints also decrease the chalcogenide displacement vectors with L1 > L2 > L3, for all three chalcogenides. Note: the PKS vibration for **1**-L1 was split into two modes.

Incorporation of Vibronic Coupling into the Spin Ladder Energy

$$E_{\pm} = -JS(S+1) + \frac{1}{2}\left(\frac{\lambda^2}{k_{-}}\right)x_{-}^2 \pm \left[\frac{1}{2}\left(\frac{\lambda^2}{k_{-}}\right)^2 x_{-}^2 + B^2\left(S+\frac{1}{2}\right)^2\right]^{1/2}$$

For the above equation, λ (cm^{-1}/ Å) = $k_{-}(\Delta Q_{-}) = k_{-}(n^{1/2}(\Delta r))$, where Δr (Å) is the difference in metal-ligand bond lengths between oxidized and reduced monomeric subunits (an approximation of the PKS nuclear displacements), n is the coordination number of each ion, x_{-} is the dimensionless coordinate associated with the Q_{-} vibrational normal coordinate ($Q_{-} = (\lambda/k_{-})x_{-}$) having vibrational frequency ν_{-} (cm^{-1}), and $k_{-} = 4\pi^2c^2m(\nu_{-})^2$ is the force constant for nuclear distortion along this coordinate (k_n/hc gives units of cm^{-1}/Å^2, $m = \mu/6.02\times10^{23}$ mol^{-1}).[6] For complexes **1-3**, Δr = 0.1 Å, n = 4, μ_1 = 23 g/mol, μ_2 = 46 g/mol, μ_3 = 71 g/mol, and frequencies, ν_{-}, were taken from **Supplemental Table 10** (L3). The resulting values are given in **Supplemental Table 11** and used in **Supplemental Fig. 37**.

Table 11. Comparison of B, *J*, and vibronic coupling terms for complexes **1-3** with $[(Me_3tacn)_2Fe_2(OH)_3]^{2+}$ and a generalized $[Fe_2S_2]^+$ ferrodoxin.[6]

	$[Fe_2(OH)_3]^{2+}$	Fd.-$[Fe_2S_2]^+$	**1-$[Fe_2S_2]^+$**	**2-$[Fe_2Se_2]^+$**	**3-$[Fe_2Te_2]^+$**
B (cm^{-1})	1350	965	200	265	750
J (cm^{-1})	-70	-360	-55	-50	-200
λ^2/k_{-} (cm^{-1})	2590	3660	2200	1400	1650
$\|B/J\|$	19.3	2.7	3.6	5.3	3.8

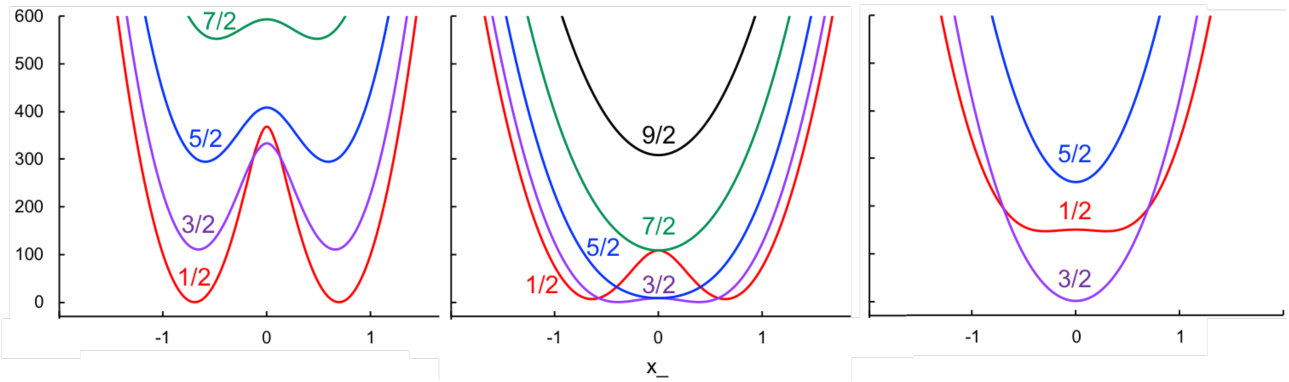

Figure 37. Ground and excited spin-state potential surfaces in the $x\_$ coordinate ($Q\_ = (\lambda/k_{-})x_{-}$, the PKS normal coordinate) calculated using above equation and parameters given in **Supplemental Table 11**, showing (a) a partially delocalized S=1/2 ground state for **1**, with a moderate thermal barrier for electron transfer (ca. 300 cm^{-1}); (b) a more compressed spin ladder of **2** with the S=1/2, 3/2, and 5/2 all very close in energy (<50 cm^{-1}), and small thermal barriers to electron transfer (<125 cm^{-1}) (note: for the purpose of generating the adiabatic potential surfaces we are assuming a single spin ladder, though experimentally **2** exhibits two spin-isomers with independent spin ladders); and (c) a fully delocalized S=3/2 ground state for **3**, with a small energy gap between the S=3/2 and S=1/2 spin states (ca. 150 cm^{-1}).

References

1 Berto, T. C. *et al.* Structural and electronic characterization of non-heme Fe(II)-nitrosyls as biomimetic models of the Fe(B) center of bacterial nitric oxide reductase. *J. Am. Chem. Soc.* **133**, 16714-16717, doi:10.1021/ja111693f (2011).

2 Albers, A. *et al.* The complete characterization of a reduced biomimetic [2Fe-2S] cluster. *Angew. Chem. Int. Ed.* **50**, 9191-9194, doi:10.1002/anie.201100727 (2011).

3 Ding, X. Q. *et al.* Exchange interactions, charge delocalization, and spin relaxation in a mixed-valence di-iron complex studied by Mössbauer spectroscopy. *J. Chem. Phys.* **99**, 6421-6428, doi:10.1063/1.465881 (1993).

4 Coropceanu, V., Brédas, J. L., Winkler, H. & Trautwein, A. X. Manifestation of the double-exchange interaction in the Mössbauer spectrum of mixed-valence systems. *J. Chem. Phys.* **116**, 8152-8158, doi:10.1063/1.1467906 (2002).

5 Girerd, J. J. Electron transfer between magnetic ions in mixed valence binuclear systems. *J. Chem. Phys.* **79**, 1766-1775, doi:10.1063/1.446021 (1983).

6 Gamelin, D. R., Bominaar, E. L., Kirk, M. L., Wieghardt, K. & Solomon, E. I. Excited-State Contributions to Ground-State Properties of Mixed-Valence Dimers: Spectral and Electronic-Structural Studies of [Fe2(OH)3(tmtacn)2]2+Related to the [Fe2S2]+Active Sites of Plant-Type Ferredoxins. *J. Am. Chem. Soc.* **118**, 8085-8097, doi:10.1021/ja9536633 (1996).

7 Bechlars, B. *et al.* High-spin ground states via electron delocalization in mixed-valence imidazolate-bridged divanadium complexes. *Nat. Chem.* **2**, 362-368, doi:10.1038/nchem.585 (2010).

8 Fu, W., Drozdzewski, P. M., Davies, M. D., Sligar, S. G. & Johnson, M. K. Resonance Raman and magnetic circular dichroism studies of reduced [2Fe-2S] proteins. *J. Biol. Chem.* **267**, 15502-15510, doi:10.1016/S0021-9258(19)49563-X (1992).

9 Subramanian, S. *et al.* Spectroscopic and redox studies of valence-delocalized [Fe2S2](+) centers in thioredoxin-like ferredoxins. *J. Am. Chem. Soc.* **137**, 4567-4580, doi:10.1021/jacs.5b01869 (2015).

10 Sharma, S., Sivalingam, K., Neese, F. & Chan, G. K. Low-energy spectrum of iron-sulfur clusters directly from many-particle quantum mechanics. *Nat. Chem.* **6**, 927-933, doi:10.1038/nchem.2041 (2014).

11 Jordanov, J., Roth, E. K. H., Fries, P. H. & Noodleman, L. Magnetic studies of the high-potential protein model [Fe4S4(S-2,4,6-(iso-Pr)3C6H2)4]- in the [Fe4S4]3+ oxidized state. *Inorg. Chem.* **29**, 4288-4292, doi:10.1021/ic00346a025 (2002).

12 Sridharan, A., Brown, A. C. & Suess, D. L. M. A Terminal Imido Complex of an Iron-Sulfur Cluster. *Angew. Chem. Int. Ed.* **60**, 12802-12806, doi:10.1002/anie.202102603 (2021).

13 Blum, H., Salerno, J. C., Prince, R. C., Leigh, J. S., Jr. & Ohnishi, T. Electron paramagnetic resonance determination of a low-lying excited state in Chromatium vinosum high-potential iron protein. *Biophys. J.* **20**, 23-31, doi:10.1016/S0006-3495(77)85534-3 (1977).

14 Castillo, R. G. *et al.* Probing Physical Oxidation State by Resonant X-ray Emission Spectroscopy: Applications to Iron Model Complexes and Nitrogenase. *Angew. Chem. Int. Ed.* **60**, 10112-10121, doi:10.1002/anie.202015669 (2021).
